# False memories in forensic psychology: do cognition and brain activity tell the same story?

**DOI:** 10.3389/fpsyg.2024.1327196

**Published:** 2024-05-16

**Authors:** Nieves Pérez-Mata, Margarita Diges

**Affiliations:** Department of Psicología Básica, Universidad Autónoma de Madrid, Madrid, Spain

**Keywords:** misinformation effect, Deese-Roediger-McDermott (DRM) paradigm, event implantation paradigm, true and false memories, event-related potentials, neuroimaging methods, repressed memories, child sexual abuse

## Abstract

One of the most important problems in forensic psychology is the impossibility of reliably discriminating between true and false memories when the only prosecution evidence comes from the memory of a witness or a victim. Unfortunately, both children and adults can be persuaded that they have been victims of past criminal acts, usually of a sexual nature. In adults, suggestion often occurs in the context of suggestive therapies based on the belief that traumatic events are repressed, while children come to believe and report events that never occurred as a result of repeated suggestive questioning. Cognitive Researchers have designed false memory paradigms (i.e., misinformation effect, Deese-Roediger-McDermott paradigm, event implantation paradigm) to first form false memories and then determine whether it is possible to reliably differentiate between false and true memories. In the present study, we review the contribution of cognitive research to the formation of false memories and the neuropsychological approaches aimed to discriminate between true and false memories. Based on these results, we analyze the applicability of the cognitive and neuropsychological evidence to the forensic setting.

## Introduction

Currently, one of the most important problems of forensic psychology is the possibility that some facts reported as crimes are the result of the use of suggestive techniques. Indeed, both children and adults can be persuaded that they have been victims of past criminal events, usually of a sexual nature. For adults, suggestion often occurs in therapeutic contexts based on the belief that the traumatic events are repressed—and therefore they cannot be remembered at present—but provoke serious symptoms in the patients (e.g., anxiety, depression, eating disorders). With the rediscovery of the repressed memory in therapy, it is possible to overcome those symptoms during therapy, which could include elements such as guided imagery, hypnosis or induction of memories (for reviews, see Lindsay and Read, 1994, 1995, 2001). Thus, the same therapy rediscovers and heals the trauma using suggestive techniques for both purposes.

Children may also come to believe, and then claim, events that never happened actually occurred as a result of repeated suggestive questions from the family, therapeutic, police or justice environment when the interrogator is convinced that sexual abuse has taken place (Garven et al., 1998; Bruck and Ceci, 1999; Wood and Garven, 2000; Ceci and Bruck, 2006). In this case, suspicion may be based on the observation of behavioral and emotional changes (omission, increase or new onset), such as nightmares, enuresis, sexualized behavior and language, apathy or hyperactivity; these changes could be considered unequivocal indicators of abuse.

The most relevant concern is that once someone firmly believes that the suggested events occurred, false and true memories can become indistinguishable (Bernstein and Loftus, 2009). However, it is obvious that in the forensic field, it is of utmost importance to distinguish them. To this end, researchers have been interested in determining whether it is possible to reliably differentiate between these two types of memories to assist in judicial decision-making.

In the laboratory, researchers have successfully generated false memories under controlled experimental conditions to examine similarities and differences between false and true memories. In the next section, the experimental paradigms that have allowed to establish the theoretical and empirical bases of how false memories are generated are described.

## Paradigms of false memories in the laboratory

The first paradigm that showed that people can believe that they have seen details not actually perceived is currently termed the *misinformation effect* (Loftus, 1975, 1996; for reviews, see Loftus, 2003, 2005). In the standard procedure, participants are visually presented with an event (e.g., a traffic accident). Next, in the experimental condition, misinformation about a number of critical items from the event (e.g., a yield sign) is introduced through a narrative or questionnaire; whereas in the control or consistent condition, the critical details refer to generic information perceived in the original event (e.g., a traffic sign) or to consistent information (e.g., a stop sign). Later, participants complete a two-choice forced recognition test in which they are instructed to base their responses on the original event observed. For the critical items, the response options are the original detail (e.g., the stop sign truly seen at the intersection) and the misleading detail introduced in the verbal postevent task (i.e., the yield sign). Participants in the misleading condition select the misleading item significantly more frequently than the original item in comparison to the participants in the control or consistent condition. Further developments of the procedure have included additional measures such as confidence estimations and reaction time (Loftus, 1996), source attribution (Lindsay and Johnson, 1989; Johnson et al., 1993), Tulving’s (1985) phenomenological judgments of remember/know (Pérez-Mata and Diges, 2007), and qualitative measures from descriptions (Schooler et al., 1986; Pérez-Mata and Diges, 2007). However, none of these additional measures have been able to successfully discriminate between true and suggested items, indicating that once participants accept the misleading details, they are unable to access the perceived details observed in the original event (Pérez-Mata et al., 2002; Loftus, 2003, 2005). Thus, this procedure has proven to be very fruitful in establishing, both theoretically and empirically, how suggested information affects the memory performance of both experimental participants and witnesses in court cases (for reviews, see Loftus, 2003, 2005; Diges, 2016).

Another paradigm that has provided evidence that it is difficult to discriminate between perceived and nonpresented items is the *Deese-Roediger-McDermott (DRM) paradigm* (Deese, 1959; Roediger and McDermott, 1995). Participants are presented with lists of words (e.g., candy, sugar, chocolate) and other semantic associates of a word that is not presented (“sweet,” usually called *critical lure*). Later, in the retrieval phase, participants often produce the critical lure (“sweet”) on a free recall test and/or recognize it in an old/new test (for reviews, see Gallo, 2010; Jou and Flores, 2013; Coburn et al., 2021). Furthermore, participants are able to give details about the critical lure supposedly presented in the list; for example, they can identify the voice that “pronounced” the false word (female or male), indicate the order in which the critical lure was presented in the list or assign a “remember” judgment to the critical lure (e.g., Roediger and McDermott, 1995; Read, 1996; Stadler et al., 1999; Pérez-Mata et al., 2002). In recall tasks, under certain extreme encoding conditions, the critical lure can be remembered up to 50% of the time, and in the recognition task, a false recognition rate of up to 80% can be achieved (e.g., under the divided attention condition for encoding the lists, Pérez-Mata et al., 2002, 2022). Additionally, when participants report the critical lure, they are unable to backtrack and recognize that the item was, in fact, suggested by themselves due to its activation during the presentation of the words of the list.

At the same time that the DRM paradigm was developed, researchers tried to take one more step and managed to implant an entire event in adult and child participants (Hyman et al., 1995; Loftus and Pickrell, 1995; Hyman and Billings, 1998; Porter et al., 2000; Mazzoni and Memon, 2003). In the *event implantation paradigm*, the researcher enlists the collaboration of a close relative of the participant who knows the exceptional events experienced by the participant in his or her childhood (e.g., hospital admission, getting lost in a shopping mall). For each participant, three true events and one false event are selected. Through several weeks, the participant is repeatedly asked to recall the events (true and false). When plausible false events are suggested (e.g., getting lost in a shopping center),^1^ approximately 15% of adults and 25% of children remember the false event in detail. These figures increase to more than 35% and 60%, respectively, when suggestive techniques such as the generation of mental images about the false event are included in the procedure (Hyman and Pentland, 1996). Furthermore, once participants explain how they experienced the false event, they are unable to realize that it did not truly happen but was the product of repeated exposure to questions about the alleged event and the use of suggestive techniques that facilitated the construction of a false detailed narrative of the event not experienced.

After more than half a century of research devoted to the study of false memories, there are two main theories about the origin of false memories. According to the *source monitoring framework* (SMF; Johnson et al., 1993), it is postulated that when participants claim to have seen the suggested detail, or in a memory test they remember or recognize the critical theme word as studied word, or when people believe that they have experienced a false event is because they erroneously attribute the suggested, the theme word, or the false experience to a perception (i.e., they saw the suggested detail, or they listened the theme word, or they lived the false event). Moreover, if the false information is plausible and fits well with the person’s previous experience and knowledge, such misattributions are more likely to occur. In fact, when the suggested detail is very blatant and the person has no way to integrate it in his or her recall of the original event, it is rejected. Likewise, when the false event supposedly experienced is very weird and the person does not have a prior knowledge where to cling to (e.g., a rectal oedema), it is unlikely that person “remember” to have lived it because it is not possible to construct a memory about that and later to remember it like a real experience. Finally, the theme word is easily recalled or recognized it because it fits perfectly into the list heard and highly likely it was activated during the presentation of the word list due to semantic-conceptual association with the words of the list (Roediger and McDermott, 1995; Read, 1996; Roediger et al., 2001).

On the other hand, according to the *fuzzy trace theory* (FTT, Brainerd and Reyna, 1998, 2002), two different memory traces encode and store information, the *verbatim* and *gist* traces. The *verbatim* traces refer to the surface and detailed features of items, and *gist* traces refer to the overall meaning of the information being processed. Verbatim traces are highly susceptible the effects of time, whereas gist traces are more resilient. Thus, false memories are more likely to occur when verbatim traces have weakened, and memory relies mainly on gist traces. In this situation, plausible suggested details are more easily integrated into the memory of the event. The theme word fits perfectly into the gist of the list heard. However, it is difficult to explain how a completely false event can be generated and remembered as a real experience when there is not a gist trace to link it to, which could enable the generation of the false memory.

Regardless of the explanation for the phenomena of false memory, the analysis of memory performance and phenomenological experiences seems to indicate that some conditions favor similarities between true and false memories and make it highly difficult to correctly attribute the origin of memories (i.e., experienced versus suggested, self-suggested or invented), both for the person who remembers and for the person who has to evaluate the memory (Schooler et al., 1986). Thus, in the forensic field, it is concluded that the main problem is identifying a false memory when it has occurred and dismantling it to attempt to recover what truly happened.

Due to this impossibility of reliably discriminating between true and false memories at a functional level of analysis, there has been increasing interest in establishing the neurological basis for distinguishing between true and false memories, with the aim of determining whether brain responses allow something that has not been possible from a behavioral, cognitive or phenomenological approach, i.e., whether brain activity would allow reliable discrimination between true and false memories. Thus, in the last two decades, there has been an increasing interest in establishing the correlates between cognitive processes and the brain activity underlying the generation of false memories (Gallo, 2010; Jou and Flores, 2013; Dennis et al., 2023). In the next section, we address the contributions of studies aiming to identify the neural correlates of false memories. We will first look at research using the recording of evoked-related-potentials (ERPs) and then present the results that have been found using traditional and more recent neuroimaging approaches. To get a complete picture of the brain activity correlated with false memories in the DRM and misinformation paradigms, it is useful to look at the results obtained with both methods of recording brain activity, since ERPs give precise information about the timing of the brain response related to cognitive processes, while neuroimaging methods are more informative about the brain locations related to those cognitive processes. Despite the different contributions that both methods have made to our understanding of brain activity and cognitive processes, studies that have focused on reviewing the results of neuroimaging research (see sections on neuroimaging studies below) have paid little attention to the results obtained with ERP methods. This paper attempts to review the main contributions of both approaches. It should be noted that this review is not a meta-analysis or mega-analysis, nor is it intended to be a quantitatively exhaustive and indiscriminate review of published works. Rather, it is a review that analyzes which false memory paradigms have been addressed by ERP and neuroimaging methods, and what impact this has had in the forensic field, where it is necessary to answer relevant questions in criminal cases.

## Neural correlates of true and false memories

At present, not all false memory paradigms are amenable to brain activity analysis due to methodological limitations. Thus, studies on the misinformation effect have been scarce and limited, and these studies occurred later than the studies using the DRM paradigm, which have been particularly fruitful in establishing correlates between cognitive processes and brain activity (Gallo, 2010; Jou and Flores, 2013; Dennis et al., 2023). However, to the best of the authors’ knowledge, no studies using the event implantation paradigm have measured the brain activity underlying the false memory generated.

Below, we will first present the components of event-related potentials (ERPs) related to memory processes. Second, we will review the main empirical findings regarding the relationships of the DRM and misinformation paradigms with ERP components. Third, we will review the findings from neuroimaging techniques with these two paradigms.

### Memory and components of event-related potentials

Classic studies recording event-related potentials (ERPs) have typically used the yes/no recognition task to measure memory because it fits very well with the ERP methodology. In these studies an old/new effect, that involves more positive waveforms for correct “old” responses to studied items than for correct “new” responses to items not presented in the encoding phase, is typically observed (Johnson, 1995; Rugg, 1995; Friedman and Johnson, 2000; Rugg and Allan, 2000; Rugg and Curran, 2007). More positive ERP waveforms for true recognition have a broad temporal and spatial distribution that is decomposed into three spatiotemporally specific effects (Friedman and Johnson, 2000; Mecklinger, 2000): an early frontomedial old/new ERP effect (the FN400) that starts at approximately 300 ms after stimulus onset and lasts approximately 200 ms (Finnigan et al., 2002; Gonsalves et al., 2005; Curran and Hancock, 2007); a later parietal old/new ERP effect, also known as the late parietal component (LPC; Curran, 2000), that starts at approximately 400 ms and lasts for several hundred milliseconds; and a late right frontal old/new ERP effect that starts at approximately 800 ms and is sustained for longer than the other two ERP effects, up to 1900 ms after stimulus onset (Wilding and Rugg, 1996; Allan et al., 1998; Mecklinger and Meinshausen, 1998; Friedman and Johnson, 2000; Hayama et al., 2008; Morcom, 2015).

These three components have been associated with different cognitive processes. The FN400 has been related to familiarity processes, the LPC to recollection processes, and the late right frontal old/new effect to postretrieval processes (Curran, 2000; Curran and Cleary, 2003).

In addition to the three components described, a fourth component has been described: the P3b, which is a subcomponent of the P300 complex. The P3b is a positive ERP component maximal over parietal recording sites between 250 and 500 ms after stimulus onset that is associated with enhanced attention to the target stimulus and with context updating (Polich, 2007).

The FN400, LPC and late right frontal effects have been systematically studied in the DRM paradigm, whereas the P3b and the LPC have been explored in the misinformation effect. Next, we summarize the main ERP findings in both paradigms. Unlike cognitive experimental research, in which the misinformation paradigm was first developed in the mid-1970s and the DRM paradigm was subsequently developed in the mid-1990s, ERP research started studying false recognition with the DRM paradigm and later adapted the misinformation paradigm to allow ERP recordings. In this case, the chronological inverse order between the paradigms is due to the greater difficulty in adapting the misinformation paradigm than the DRM paradigm to the ERP methodology. We respect this inverse order in the next sections.

### ERP components and DRM paradigm

To date, studies have explored a wide variety of independent variables, but the results have been inconsistent with respect to any of the three ERP components examined.

#### FN400 old/new effect

This early component (300–500 ms) is specifically sensitive to conceptual/semantic information (Voss and Paller, 2009) and results in a feeling of familiarity (Mecklinger, 2000). In this sense, when this component has been explored in the DRM paradigm, it has been associated with true and false memories derived from recognition based on familiarity processes. Thus, researchers have manipulated variables that, at some level, are sensitive to recognition based on familiarity processes.

Curran et al. (2001) divided participants into good and poor performers^2^ with the DRM lists, but they did not find significant differences in the FN400 component between true and false memories for either of these two groups. In contrast, Favre et al. (2020) found a lower amplitude of the FN400 component for poor performers than for good performers.

Beato et al. (2012) manipulated the depth of processing in the encoding of DRM lists. They failed to find a processing depth effect on the FN400 component, such that true and false memories based on familiarity did not differ according to whether participants had processed the presented words under shallow or deep encoding conditions.

However, Chen et al. (2008) found more positive ERPs for true and false memories than for the correct rejections of new words when critical lures were preceded by related words in the recognition test. This effect was not observed when the lures were not preceded by related words, indicating that the presence of related words preceding the lures seemed to enhance familiarity-based recognition decisions.

Boldini et al. (2013) found an overall FN400 effect for both studied words and critical lures compared to the correct rejection of new words, but surprisingly, they did not find the expected modality-matching effect in this ERP component. Specifically, they manipulated the correspondence of item presentation modality between the study and test phases (visual in both phases versus auditory in the study phase and visual in the test phase). This result seemed to indicate that familiarity, indexed by the FN400 component, is an “amodal” process (Curran and Dien, 2003) instead of relying on modality matching. Boldini et al. (2013) also suggested that semantic information may play a role in the early stages of recognition memory.

In an attempt to explore the processes involved in both committing and avoiding false memories, Cadavid and Beato (2016) compared true recognition, false recognition, and correct rejections of related lures, in addition to the correct rejections of new words. As predicted by the authors, the FN400 effect was present for true recognition, false recognition, and correct rejections of related lures compared to correct rejections of new words because the related lures shared associative and semantic features with studied words; therefore, the recognition decisions could be based on familiarity emphasized by the semantic relationship between studied words and critical lures.

#### Partial old/new effect (the LPC)

This later component (400–800 ms) is observed in central and especially parietal electrode sites and has been related to the recall of details (e.g., Allan et al., 1998; Curran, 2000) and to consciously controlled recollection of item-specific information from the encoding phase (Paller and Kutas, 1992; Wilding and Rugg, 1996; Düzel et al., 1997; Rugg and Curran, 2007; and for reviews, see Johnson, 1995; Rugg, 1995; Allan et al., 1998). Unlike the FN400 component, higher amplitudes are observed when variables requiring recollection processing are involved in recognition (Paller and Kutas, 1992; Paller et al., 1995; Rugg et al., 1995). Thus, the LPC is mainly observed when specific details of stimulus encoding are correctly remembered, and larger parietal old/new effects are associated with remembering than with knowing in the Tulving’s (1985) judgments (Düzel et al., 1997; Rugg et al., 1998; Smith, 2003). However, in the DRM paradigm, inconsistent results have been found for the LPC component.

Curran et al. (2001) differentiated between poor and good performers and unexpectedly found that only the poor performers exhibited more positive parietal waveforms for true recognition than for false recognition. Beato et al. (2012) manipulated the level of processing at encoding, but they did not find a differential effect of this variable on the LPC for either true recognition or false recognition; however, they found that, overall, the left parietal waveforms were more positive for true and false recognition than for correct rejections of new items. Similarly, Boldini et al. (2013) observed the LPC for both the words studied and the critical lures but no detectable differences as a function of study-test presentation modality matching (visual–visual versus auditory–visual) in true and false recognition. Cadavid and Beato (2016) also found the parietal old/new effect for true and false recognition but not for correct rejections of related lures. In fact, there seemed to be a continuum of brain activation underlying the recollection processes among hits of studied words, false alarms of critical lures, correct rejections of critical lures, and correct rejections of unrelated new words (Kurkela and Dennis, 2016) because parietal activation was higher for studied words and false alarms of critical lures than for correct rejections of critical lures and new words, but at the same time parietal activation was higher for correct rejections of critical lures than of new words. However, Wiese and Daum (2006) found more positive amplitudes for true recognition and false recognition than for correct rejections of related lures or new words, and in addition, ERPs for false recognition were similar to those for correct rejections of related lures and new words at the frontal electrodes.

#### Late right frontal old/new effect

This component (800–1900 ms) has been related to strategic retrieval efforts (Johnson et al., 1997; Wilding and Rugg, 1997a,b; Allan et al., 1998; Ranganath and Paller, 2000; Wilding, 2000) and to postretrieval monitoring processes involved in decision-making (Hayama et al., 2008). Thus, some authors have suggested that this late postretrieval effect reflects monitoring processes (Wilding and Rugg, 1996; Senkfor and Van Petten, 1998; Ranganath and Paller, 2000; Curran et al., 2001; Kuo and Van Petten, 2006, 2008; Cruse and Wilding, 2009; Wolk et al., 2009; Boldini et al., 2013). However, the pattern of results in the DRM paradigm is inconsistent.

Curran et al. (2001) found that good performers, compared to poor performers, showed a right frontal ERP for true and false memories related to the correct rejection of new words, and the authors interpreted this brain activity as a reflection of the postretrieval evaluation processes used by participants with good behavioral discrimination between true and false recognition. Johnson et al. (1997) also found more positive waveforms for true memories than for false memories when the words were presented in blocks rather than a random order in the recognition test. In the blocked condition, participants appeared to employ more sophisticated evaluation processes that allowed them to discriminate correctly between old words and critical lures. Additionally, Beato et al. (2012) suggested that a more positive late right frontal effect for true and false recognition of items encoded in shallow-level processing conditions than in deep-level conditions indicated that items in shallow conditions might be represented in a weak memory trace and, consequently, might require more retrieval effort and/or monitoring assessment compared to items encoded in deep conditions. Similarly, Boldini et al. (2013) observed more positive waveforms of the late right frontal effect for true and false recognition than for new words; moreover, they observed an influence of the modality of presentation. Thus, in the study-test modality-matched condition (visual–visual), the ERPs elicited were significantly more positive than those in the modality-mismatch condition (auditory–visual). Thus, sensory modality matching between the study and test time points appeared to play a role in brain activity related to monitoring processes. However, Cadavid and Beato (2016) observed a late right frontal old/new effect for true and false recognition but not for correct rejections of related lures, which could be an indication that participants “remembered” the critical lures as if they had actually been presented in the encoding phase of the word lists and, consequently, had become indistinguishable from true recollections.

Actually, regard to this component there is a quite sophisticated cognitive process that shows up when it is required a more strategic and demanding post-retrieval analysis (Curran, 2000; Curran and Cleary, 2003; Morcom, 2015). For example, to reject items closely associated with heard words (“I did not hear it in the list”) might require a more strategic and demanding post-retrieval analysis, which could involve recall-to-reject processes, although the evidence was rather weak and inconclusive in this respect (Curran, 2000; Curran and Cleary, 2003; Morcom, 2015).

It is obvious that it is difficult to extract an empirical pattern from ERP activity results that clarifies the boundary conditions of discrimination between false and true memories of semantically related words to establish minimally reliable conditions for discrimination. In fact, there seems to be more consistency in the patterns traditionally observed at the behavioral, cognitive and phenomenological levels than at the neural level examined through ERP activity. It is also obvious that part of this inconsistency is due to the methodological variability employed in ERP studies, i.e., differences in the material used (Arndt, 2012; Cadavid and Beato, 2016), the modality of stimulus (list) presentation (e.g., Johnson et al., 1997; Curran et al., 2001; Boldini et al., 2013), and the recording of brain activity and its subsequent analysis (Kurkela and Dennis, 2016; Toglia et al., 2022). Unfortunately, it is currently difficult to overcome this variability and the corresponding inconsistency in the pattern of results.

### ERP components and the misinformation paradigm

In this paradigm, two main components have been examined: the LPC and the Pb3. The FN400 does not apply in this case because participants have been exposed to both the perceived details from the original event and the misleading details from the verbal postevent task, so both types of items are familiar to them. Therefore, a component that reflects familiarity processes, such as the FN400, cannot discriminate between true and suggested memories. However, the late right frontal old/new effect has not been extensively studied in the misinformation effect paradigm; only in two studies a late slow frontal positive wave has been analyzed (Kiat and Belli, 2017; Volz et al., 2019).

The LPC is a component associated with recollection processes, and as such, it has been proposed that at the retrieval phase, the LPC evoked by the perceived details will be more positive than the LPC evoked by the suggested details (Meek et al., 2013; Kiat and Belli, 2017; Volz et al., 2019). More specifically, Kiat and Belli (2017) proposed a continuum where more positive LPC amplitudes are associated with the perceived details followed by misinformation rejections (because rejections could be based on the retrieval of information contradictory to the perceptual events), while the lowest amplitudes are associated with the acceptance of misinformation at the recognition test.

However, the P3b (250–500 ms), a subcomponent of the P300 complex (which is maximal over parietal recording sites), is associated with enhanced focal attention for a target stimulus (Polich, 2007). In fact, it has been associated with memory operations involved in assessing the degree to which incoming targets match (or do not match) internal preactivated representations in working memory for subsequent action-taking. Regarding episodic memory, this component plays a key role in concealed memory detection research (e.g., Gamer and Berti, 2012; Ganis and Schendan, 2013; Meixner and Rosenfeld, 2014). In these studies, probes linked with true episodic memories are associated with higher P3b amplitudes than those linked with neutral (Gamer and Berti, 2012; Meixner and Rosenfeld, 2014), nonepisodic (Ganis and Schendan, 2013), or irrelevant probes (Ambach et al., 2010; Rosenfeld and Labkovsky, 2010; Matsuda et al., 2011; Meek et al., 2013).

Given the characteristics of the P3b component, it is expected that the acceptance of misleading details is associated with lower P3b amplitudes than those of perceived details; however, this does not exclude the possibility that, as the misinformation is presented with the narrative, this type of detail could result in a context-matched signal largely equivalent to that for perceived details presented in the original event (Meek et al., 2013; Kiat and Belli, 2017). In contrast, for misinformation rejections, a higher P3b amplitude is proposed, although it is unclear if the discrepancy detection response would exceed the context-matched response for perceived details (Kiat and Belli, 2017). However, based on Meek et al. (2013) and Volz et al. (2019), it has been proposed that to the extent that misleading information distorts the memory of the original event, the perceived information loses its meaning, while misleading information gains meaning; therefore, the amplitude of the P3b component would be more positive for the accepted misleading details and for the originally perceived details than the rejections of the original and control details.

Kiat and Belli (2017) found a continuum of P3b amplitudes because they observed a more positive P3b amplitude for the recognition of accurate events (statistically significant) and for misinformation rejections (marginally significant) than for misinformation acceptance. They also found that LPC amplitudes were significantly higher for true memories than for misleading memories and descriptively higher for misinformation rejections than for misinformation acceptances. However, Volz et al. (2019) did not find significant differences between suggested and true memories in the P3b or LPC. For both components, more positive amplitudes were associated with true and suggested memories rather than with correct rejections. Last, in the study of Meek et al. (2013), the same pattern of results was found for both the P3b and LPC. Specifically, as predicted by the authors, smaller amplitudes were observed for misinformation details than for consistent information, but contrary to expectations, in the condition in which participants had to lie about the misleading information, they showed consistently lower P3b amplitudes. Meek et al. (2013) attributed this result to the fact that this condition, in which participants had to discriminate between misleading and true information and lie about the misleading information, was the most difficult experimental condition because it imposed the highest memory load and, therefore, could affect P3b amplitudes. Furthermore, Meek et al. (2013) found smaller LPC amplitudes for misinformation than for consistent information, so less recollective activity was associated with misinformation than with consistent information.

Furthermore, Kiat and Belli (2017) and Volz et al. (2019) analyzed a frontal slow wave (approximately 500–800 ms). Kiat and Belli did not find differences in this component, whereas Volz et al. found significant differences in this component between suggested and true memories but not between true memories and correct rejections or between suggested memories and correct rejections. Thus, it is not clear if the three types of items that were analyzed in these studies (true or consistent details, misinformation details, and correct rejections) systematically reflected similar or different memory retrieval efforts when participants attempted to respond in the recognition task.

Once again, inconsistent empirical patterns have been observed and methodological differences could be partly responsible for the discrepancies observed. The study conducted by Kiat and Belli (2017) generated several events to provide a large number of independent critical true and suggested details for the recognition task; however, in the other two studies, the recognition task asked about the same critical items (true and suggested) multiple time to obtain the minimum number of trials required to allow the subsequent analysis of ERP activity. Obviously, asking for the same item repeatedly can lead to a change in the answer, as well as the inability to guarantee independence between the answers given by the same participant. Furthermore, Meek et al. (2013) used short retention intervals between the three phases (the original event, postevent narrative and recognition task), although the most important limitation of their study was that the authors used the misinformation paradigm modified by McCloskey and Zaragoza (1985). This procedure was previously proven to be inadequate for studying the misinformation phenomenon, to the point that Zaragoza herself abandoned this procedure in the 1990s; it has not been used since in cognitive studies because it is a noninformative procedure, both theoretically and methodologically. Furthermore, the study by Meek et al. (2013) also included a deception condition in which participants had to deliberately lie (or not) in the recognition test; therefore, it is not easy to disentangle the misinformation effect from the effects of intentionally lying in the deception condition.

Next, the results of neuroimaging studies are described. Excellent and extensive literature reviews of neuroimaging studies have been published that assess false memories (Abe, 2012; Johnson et al., 2012; Schacter et al., 2012; Dennis et al., 2015; Kurkela and Dennis, 2016; Van de Ven et al., 2017; Dennis et al., 2023). Some authors (e.g., Dennis et al., 2015; Kurkela and Dennis, 2016; Dennis et al., 2023) have conducted a meta-analysis or strictly quantitative review of the neuroimaging literature regarding false memory formation, collapsing across studies using different stimuli, baseline and experimental paradigms. The aim of these reviews is to identify consistent brain regions activated when a false memory is generated and compare them with the regions activated for true memories, regardless of the type of procedure employed. Thus, these reviews can indicate which brain regions are most active during the encoding and/or retrieval phases for false memories as a whole. However, such reviews do not allow us to know which regions are specifically relevant for each particular paradigm and for each variable manipulated in those paradigms (for a review, see Dennis et al., 2023). As indicated above, we do not present a review of previous reviews, or a meta-analysis or a mega-analysis, but rather our interest is to examine which neuroimaging findings may be useful in the forensic context, and to this end it is necessary to present results for each of the false memory paradigms that have contributed to the understanding of memory errors in witnesses and victims. Thus, next, we present the results of the DRM paradigm to continue with the results of the misinformation paradigm.

### Neuroimaging studies and the DRM paradigm

The neuroimaging methods used with the DRM paradigm have been varied, including variation in the brain structures examined and methodologies used, so it is difficult to obtain a clear picture of the relationships between brain activity and cognitive functioning and we have a somewhat vague patchwork of relationships.

In the majority of neuroimaging studies, brain activity has been recorded during the retrieval phase, i.e., when participants are completing the memory test and the memory error itself occurs. First, several studies have consistently shown that the sensory cortex is mostly linked to true memories rather than to false memories. In a pioneering work, Schacter et al. (1996) used positron emission tomography (PET) and reported similarities and differences in brain activation between these two types of memory. Compared with a common baseline condition, both true and false recognition were associated with increased blow flow in regions implicated in memory processing: the anterior and dorsolateral prefrontal cortex (PFC), medial parietal cortex and medial temporal regions. They also found that the left temporoparietal cortex (Brodmann’s area [BA] 42/22/40), an area associated with auditory processing, showed greater activation during true recognition than during false recognition. Schacter et al. interpreted these findings in the context of the sensory reactivation hypothesis (Schacter and Slotnick, 2004; Schacter et al., 2012). Because participants had heard true, but not false, targets during the auditory encoding phase, the activation of the left temporoparietal cortex for true recognition might be a sensory signature that reflects memory traces for auditory aspects of previously studied words. Later, Abe et al. (2008) used a variant of the DRM paradigm and reported greater activity for true recognition than for false recognition in the left temporal and parietal cortices.

One methodological limitation of PET imaging studies is that stimuli from different conditions—studied words, critical lures and new words—have to be presented in separate blocks in the recognition test. Event-related functional magnetic resonance imaging (fMRI) methods overcome this limitation and allow the intermixing of items from different conditions. However, in an fMRI study, Schacter et al. (1997) failed to replicate the greater activation in the left temporoparietal area for true memories than for false memories.

To test the sensory reactivation hypothesis more directly, Cabeza et al. (2001) used study conditions that promoted the encoding of sensory information. Participants viewed videotapes in which a male voice spoke half the words and a female voice spoke the other half; they were instructed to remember the presented words and which speaker pronounced them. The authors believed that these encoding conditions would encourage the encoding of sensory information (i.e., speakers’ voices and faces linked to each presented word). The results showed that the parahippocampal gyrus, a region within the medial temporal lobe (MTL) linked to the processing of contextual information, and the left parietal cortex (BA 39/40), a region implicated in auditory word processing, were activated during true recognition but not during false recognition. Cabeza et al. interpreted this activation as reflecting the recovery of sensory information during the encoding of word lists that were accompanied by rich contextual and sensory information.

Currently, left temporoparietal activity is regarded as a reliable neural signature of true recognition (for updated reviews of this issue, see Dennis et al., 2015, 2023).

Second, other regions that have shown differences in activation between true and false memories are located in the medial temporal lobe (MTL). For example, Cabeza et al. (2001) found that the anterior hippocampus exhibited similar activation for both memories, whereas the posterior parahippocampal gyrus (PHG) showed greater activity for true memories than for false memories. According to Cabeza et al., the anterior regions reflect recovery of semantic information, which would support both types of memory, whereas the posterior PHG is connected to sensory cortices and reflects retrieval of sensory information specific to true memories but not to false memories. In contrast, Schacter et al. (1997) did not find differences in MTL activation between true and false recognition.

Subsequent studies have further examined MTL structures to establish patterns of similarities and differences between true and false memories in the DRM paradigm. Chadwick et al. (2016) used fMRI and representational similarity analysis to obtain measures of overlap, showing that the only significant cluster of positive correlations between neural overlap and the likelihood of a DRM illusion was in the left anterior temporal lobe (ATL). Thus, concrete patterns of ATL activity, assumed to reflect the degree of semantic similarity between concepts, successfully predicted false recognition, providing empirical support for the idea that the ATL plays a critical role in the formation of semantically driven memory illusions and acts as an amodal semantic hub (for more details, see Chadwick et al., 2016; Díez et al., 2017). Chadwick et al.’s finding represented a breakthrough in understanding the involvement of the ATL in semantic processing and the nature of semantic representations, but the results provided only correlational evidence. However, three studies (Boggio et al., 2009; Gallate et al., 2009; Díez et al., 2017) used transcranial direct current stimulation (tDCS), a noninvasive brain stimulation method that allows the establishment of casual relationships (not mere correlations) between brain activity and cognitive processes, as in studies using fMRI.^3^ The main finding from these studies was the differential effect of tDCS of the left ATL on false memory formation. A substantial reduction in false memory was observed after anodal stimulation relative to that in the sham condition, indicating that semantic relation-based memory illusions were differentially affected by anodal stimulation. Thus, anodal stimulation appeared to hamper the processes underlying the establishment of an integrated conceptual network during the encoding of associated lists (Díez et al., 2017).

A third region involved in the differentiation of true and false memories in the DRM paradigm is the prefrontal cortex (PFC). Several studies have reported increased activity in the bilateral prefrontal cortex for false memories compared to true memories (e.g., Schacter et al., 1996, 1997; Cabeza et al., 2001). Schacter et al. (1996) found that the right dorsolateral and anterior PFC exhibited greater activation for false recognition than for true recognition, and the authors concluded that this differential activation may reflect the need for increased retrieval monitoring and evaluation associated with the strong familiarity evoked by the false memory (i.e., the critical lure). ERP studies have also found that frontal region activations differed between true and false memories, with false retrieval engaging more monitoring and evaluation processes (e.g., Curran et al., 2001; Beato et al., 2012; Boldini et al., 2013). Thus, it appears that when critical lures are semantically related to studied words, the semantic gist may evoke a sense of familiarity that is strong enough to form the basis of a false memory (Kim and Cabeza, 2007a; Dennis et al., 2015).

More specifically, Toglia et al. (2022, Study 2) recently used functional near-infrared spectroscopy (fNIRS)^4^ to measure the activity of the left and right prefrontal cortices in three brain regions: the anterior (APFC), dorsolateral (DLPFC) and ventrolateral (VLPFC) prefrontal cortices. The results indicated that false memories (critical lures) and incorrectly believing that other new words were old elicited more activity in the PFC overall than true memories (studied words) and correct rejections of new words. More specifically, the PFC region most activated by true memories was the left DLPFC, while false memories increased activation in both the left and right DLPFC. Toglia et al. argued that increased DLPFC activity might reflect monitoring decisions when participants evaluate whether a critical lure was on the list because this type of decision could require more cognitive effort due to the semantic relationship of the critical lure with the studied words. Last, Toglia et al. (2022) found increased activity in the left APFC, similar to the findings of Schacter et al. (1996) PET results.

Additionally, researchers are interested in deeply exploring the role of parietal structures in true and false memories. McDermott et al. (2017) found that overall, there was greater parietal activation for true and false memories than for correct rejections. More specifically, this pattern appeared in the lateral and medial surfaces, dorsal surfaces, right precuneus and right posterior intraparietal lobule/dorsal angular gyrus (IPL/dAG). Additionally, activity foci were observed in the precuneus, the midcingulate cortex (MCC) and the left posterior inferior parietal lobule/dorsal angular gyrus (pIPL/dAG). Thus, true and false memories activated the parietal memory network (PMN) to a similar extent and did so to a greater extent than correct rejections of new words. Moreover, when the regions outside of the PMN were examined, similar activity for true and false memories (and different activity from that elicited by correct rejections of new words) was found in the following regions: the left insula and medial frontal region in the cingulo-opercular and salience networks, bilateral inferior parietal sulcus, left middle frontal gyrus and left anterior frontal cortex (in or near the frontoparietal network), bilateral angular gyrus (more pronounced on the left side) and posterior cingulate.

Finally, when McDermott et al. (2017) directly compared the brain activation of true and false memories, they did not observe many differences. Thus, greater activity for true memories than false memories predominantly occurred in the left hemisphere, except for activation of the intraparietal sulcus (IPS) in the dorsal attentional network, which exhibited bilateral activity. Additionally, the left ventromedial occipitotemporal cortex and hand/somatomotor cortex exhibited greater activity for true memories than for false memories. These results were unexpected because regions demonstrating differences between true and false memories generally exhibit less activation for the false memories; however, these regions are in control networks (e.g., the left dorsal frontal and bilateral IPS). In fact, greater cognitive control (and therefore greater activity in control regions) is expected during the false memory condition, as other research has shown (e.g., Schacter et al., 1996; Toglia et al., 2022).

Thus, in light of the results obtained, McDermott et al. (2017) concluded that this network clearly showed that the objective nature of an item (i.e., old or new) was not necessarily the relevant feature of activation levels; rather, the subjective nature of the items seemed more relevant to determining brain activation.

Pergolizzi and Chua (2015) were also interested in the role played by the parietal cortex but, more specifically, in the role played by the posterior parietal cortex (PPC). They used the noninvasive brain tDCS method. In two experiments, Pergolizzi and Chua (2015) predicted that tDCS over the lateral PPC during memory retrieval would lead to greater false recognition and alteration of subjective recollection or confidence than in the sham tDCS condition. The results were in the direction expected since active stimulation over the parietal cortex led to increased false recognition rates. Pergolizzi and Chua interpreted this result due to the parietal cortex playing a causal role in recognition memory, especially in processes that could lead to false recognition. Even so, Pergolizzi and Chua (2015) emphasized that tDCS parameters can lead to altered excitability across large areas of the parietal cortex spanning both hemispheres, so in their study it was not possible to conclude which specific areas of the parietal cortex played a causal role in recognition memory.

Last, in a more recent study, Gatti et al. (2021) investigated whether the cerebellum is causally involved in semantic memory by conducting two experiments using transcranial magnetic stimulation (TMS) during the recognition test. TMS was administered over the right posterior cerebellum. Gatti et al. chose this region because verbal and semantic processing tends to be right-lateralized in the cerebellum, and associative models have proposed that semantic memory is organized and aligns with language (for more details, see Gatti et al., 2021). With this in mind, Gatti et al. expected that right cerebellar TMS would affect participants’ ability to discriminate words related to the studied words but not unrelated words. The results from Experiment 1 showed that cerebellar TMS selectively affected participants’ ability to discriminate the critical lures without affecting the ability to discriminate unrelated words. Furthermore, in Experiment 2, the hypothesis that cerebellar involvement in semantic memory follows a semantic gradient of association was tested by including weakly related lures in addition to unrelated words and critical lures. It was found that the higher the semantic association between new and studied words was, the greater the memory impairment caused by TMS. Cerebellar TMS impaired participants’ ability to correctly discriminate studied words from new words, resulting in a higher false alarm rate. Gatti et al. interpreted the result of the second experiment as representing an increased activation of associative links in semantic memory. Hence, after administering TMS over the right cerebellum during the recognition phase of the DRM, Gatti et al. found that cerebellar involvement in semantic memory followed a semantic gradient of association, with TMS inducing greater impairment in identifying critical lures semantically related to the studied words. Thus, it appears that the right cerebellum may be causally involved in the retrieval of semantic associations.

In summary, in the neuroimaging approach incorporating the DRM paradigm, overlaps and differences in brain activity recorded during the recovery phase have been found (for an updated review, see Dennis et al., 2023). Specifically, a large-scale neural overlap between the two types of memories within the bilateral frontal and parietal regions (Schacter et al., 1997; McDermott et al., 2017), bilateral caudate and insula (McDermott et al., 2017), lateral temporal cortex (Cabeza et al., 2001; McDermott et al., 2017), and ventral visual regions (McDermott et al., 2017) has been observed. Additionally, overlap across core memory regions in the MTL, including the hippocampus and PHG, has been observed (Schacter et al., 1996, 1997; Cabeza et al., 2001). This overlap may indicate that false memories rely heavily on brain mechanisms similar to those involved in true memories during the retrieval processes and that both types of memories have similar properties because the studied items and the critical lures share associative and semantic content.

However, it has been observed that the primary sensory cortex plays a critical role in the detection of true memories, whereas false memories are linked to increased activity in the frontal–parietal cortices when comparing the accuracy of memory responses, both in the form of true memories and correct rejections of critical lures (Schacter et al., 1996, 1997; Cabeza et al., 2001; Kim and Cabeza, 2007b). Furthermore, several studies have demonstrated that MTL activity is associated with false memory retrieval (Schacter et al., 1997; Cabeza et al., 2001; Kim and Cabeza, 2007b), although this activity did not substitute for the activity observed for true memories. Rather, the interaction between MTL activity and processing with other components of the retrieval network is critical to the occurrence of false memories (Boggio et al., 2009; Gallate et al., 2009; Chadwick et al., 2016; Díez et al., 2017; Dennis et al., 2023).

On the other hand, examinations of the role of encoding in subsequent memory errors have stressed the importance of frontal, MTL, medial temporal gyrus (MTG) and sensory activation. In a modified DRM paradigm, Kim and Cabeza (2007a) found that regions involved in semantic elaboration (left ventromedial and dorsomedial PFC) and conscious item processing (bilateral occipitotemporal and occipitoparietal cortex) were related to both true and false memory formation, but true memory formation was only associated with greater activity in the PHG and early visual cortex (BA 18/17). Kim and Cabeza (2007a) concluded that when richer, more fine-gained, encoding representations are formed, this leads to a stronger retrieval trace able to endorse targets and reject lures.

Finally, when representational similarity analysis (RSA), including encoding-retrieval similarity analysis (ERS), has been conducted in false memory research, the multivariate analyzes have been applied to detect the overlap (or correlation) of neural information between encoding and retrieval. These studies further highlight the fundamental importance of the visual cortex in differentiating true memories from false memories. Therefore, greater neural similarity (ERS) in occipital regions is linked to the retrieval of true memories rather than erroneous memories (Ye et al., 2016; Zhu et al., 2019). In this line, Ye et al. (2016) found that ERS within the lingual cortex was greater for true memories than false memories, and Zhu et al. (2019) observed greater ERS associated with true memories than false memories in lateral occipital regions. These findings support and extend the previous univariate findings of greater sensory activation for true memories than for false memories during retrieval (Schacter et al., 1996; Cabeza et al., 2001).

Furthermore, ERS analyzes have also highlighted the role of frontoparietal regions (Ye et al., 2016; Zhu et al., 2019). Ye et al. (2016) found that global encoding-retrieval similarity within the lateral parietal cortex supported more general memory retrieval (i.e., both true and false memories), with the magnitude of ERS in this region correlated with lure relatedness (e.g., semantic similarity). The authors also found that the relationship between increased ERS in the parietal cortex and decreased ERS in the occipital cortex for lure trials was correlated with frontal processes. Zhu et al. (2019) also observed similarities of encoding and retrieval for false memories but not correct rejections in the occipital and frontal cortices. Taken together, the above findings support the idea that sensory cortices recapitulate less information for a lure than for a target during evaluation at the time of retrieval, thus leaving degraded or incomplete memory traces that contribute to memory error. At the same time, the frontal and parietal cortex appeared engaged in *top-down processing* in the presence of novel lure stimuli, both attending to the new features and engaging in conflict monitoring processes.

Thus, in line with the findings of the retrieval studies, both the encoding evidence and ERS evidence highlight the need for a strongly encoded sensory representation of the studied information, followed by the ability to retrieve or reactivate this presentation when making memory decisions. Research across a number of analysis methods points to the need for a strong correspondence in memory representation between encoding and retrieval, supporting both higher hit and lower false alarm rates. This evidence is consistent with the sensory reactivation theory of memory that has been investigated for decades in memory research. This perspective is not new, as it is reminiscent of Tulving’s approach to the importance of retrieval cues and the reestablishment of coding conditions at the time of retrieval to allow successful retrieval (Tulving and Osler, 1968; Tulving and Thomson, 1973).

The misinformation paradigm has also been adapted to incorporate neuroimaging methodologies used to compare the neural correlates of the acceptance of misleading information with those of correctly recalling the details from the original event, although research is scare due to the difficulties of adapting the misinformation paradigm to the requirements of this type of methodology.

### Neuroimaging studies and the misinformation paradigm

Okado and Stark (2005) assessed neural activity during both the presentation of the original event and the introduction of the misinformation. They found that activity in the left hippocampus and left perirhinal cortex predicted whether the original or suggested information would be selected on a subsequent forced-choice recognition test. During the original event (i.e., encoding phase), activity in the left parahippocampal gyrus and perirhinal cortex was greater for true memories than for suggested memories, whereas during the introduction of the misinformation (i.e., retention interval), activity in the left hippocampus tail was greater for suggested memories than for true memories. Additionally, the activity in the right dentate gyrus and body, left hippocampus body and left parahippocampal cortex was greater for true memories than for false memories during the introduction of misinformation. These findings suggest that the encoding processes in the MTL system are an important determinant for true and false memory outcomes in the misinformation paradigm.

Later, Stark et al. (2010) found that true (visually perceived) memories were associated with greater activity than suggested memories in early visual cortex regions (BA 17/18), including the striate cortex, whereas misinformation memories were associated with increased activity in only the left superior temporal gyrus of the auditory cortex (BA 22/42; note that in this study, the postevent narrative was presented in the auditory modality), even when they were falsely judged as coming from a visually presented source. Stark et al. attributed the increased sensory cortex activity in true memories to the retrieval of sensory information associated with the presentation of the original information. Clearly, the results were in line with the sensory reactivation hypothesis.

Using a modified version of the misinformation procedure from the Okado and Stark (2005) study, Baym and Gonsalves (2010) examined how the presence of verbal misinformation and visual imagery may affect the formation of false memories. They found that greater activity in the regions responsible for visual processing (occipital and temporal [fusiform gyrus] cortex) during the presentation of the original event predicted subsequent true memories rather than suggested memories, indicating that differences in encoding may contribute to later susceptibility to misinformation. However, activity in the right hippocampus or bilateral parahippocampus during the original event was greater for both true and suggested memories than for forgotten items. Baym and Gonsalves interpreted this finding as reflecting the encoding of general contextual information and that susceptibility to misleading information was more likely when general contextual information was encoded rather than object-specific details, i.e., when a gist representation of the event was prioritized. In addition, the formation of false memories seemed to require that at least some of the information from the original event was encoded so that misinformation would affect subsequent recall.

Edelson et al. (2011), in a variation of the misinformation paradigm, attempted to elucidate the effects of social conformity on the formation of transient and persistent memory distortion. In their study, neural activity was recorded during the misinformation phase with fMRI. Edelson et al. found that persistent memory errors were associated with increased activity in the bilateral MTL compared to no-answer trials or trials in which participants corrected their answers in the final memory test. Thus, it appears that misinformation, which caused persistent memory distortion, resulted in memory updating, with additional encoding processes supported by the MTL.

Later, in 2017, Gordon et al. published an article on the “continued influence effect of misinformation” (CIEM). This phenomenon involves discredited information that continues to influence the beliefs and reasoning of people even after that information has been retracted (for reviews of this phenomenon, see Lewandowsky et al., 2012; Schwarz et al., 2016). Gordon et al. (2017) found that the same piece of information was processed differently in the right precuneus/posterior cingulate cortex (PrC/PCC) depending on whether prior information was retracted. Reductions in PrC activity have been linked to integration difficulties (e.g., Lahnakoski et al., 2017). Therefore, less integration would be expected when there is retraction than when there is no retraction, and consequently less activity would be observed in PrC/PCC regions.

In summary, although some of the results have reported overlapping activity associated with true and suggested memory formation (e.g., Baym and Gonsalves, 2010), most studies have consistently shown differences in the neural correlates of both memories; unfortunately, the variation among studies does not allow us to make firm conclusions about the encoding mechanisms underlying memory distortion.

Thus, in general, data from neuroimaging studies do not seem to establish clear and consistent differences or similarities between false and true memories regarding the brain regions that could be involved in the formation of these two types of memories. Part of the problem may lie in the fact that there is substantial variety and complexity of the involved cognitive processes when memories are formed and subsequently retrieved, whether it is true or false, and we currently do not understand or cannot capture this complexity through recordings of brain activity, at least for the time being. Indeed, there is debate about how to interpret neuroimaging findings and draw conclusions from them (Aguirre et al., 2003; Poldrack, 2006, 2011). The classic strategy in neuroimaging research has been to manipulate a specific psychological function and identify the localization of this manipulation on brain activity, i.e., a ‘forward inference’ strategy (Henson, 2005). However, this strategy has not always been followed, and a remarkable number of studies have used the observed brain activation to infer cognitive processing, i.e., a criticized “backward inference” strategy (Aguirre et al., 2003; Poldrack, 2006). This debate is beyond the scope of this paper, but whatever strategy is used, neuroimaging data (such as ERP data) are inherently correlational, and any significant association between brain activity and cognitive processes does not imply causality. Indeed, studies have been more successful in establishing relationships between broad cognitive processes (e.g., recognition memory) and brain regions (e.g., parietal structures), but research has been less successful in establishing clear correlations between finer-grained processes and brain regions (Poldrack, 2011) and, by the moment, it seems that there is an unbridgeable gap between what happens at the level of brain activity and what happens at the cognitive-phenomenological level; thus, there is not always a direct brain signature for true and false memories. In this context, Poldrack (2011) suggests that researchers should use all valid strategies available as useful methods for generating hypotheses without losing sight of the fact that they are to be tested and not taken as evidence in themselves.

Thus, currently, cognitive studies have been established boundary conditions for illusory memories from DRM lists and for misinformation effects (Stadler et al., 1999; Loftus, 2003, 2005; Diges, 2016). In contrast, ERP and neuroimaging studies are far from establishing systematic patterns,^5^ both within and between paradigms. Additionally, we note the differences between the two experimental paradigms. On the one hand, the DRM paradigm shows an autosuggestion phenomenon caused by the intrinsic characteristics of the material used, i.e., the close semantic and associative relationship between the words presented in the study lists (e.g., candy, sugar, chocolate) and the “phantom” word not presented (“sweet”). It is therefore a false memory based on the semantic and conceptual knowledge of the participants and does not involve the explicit presentation of false information. In contrast, the misinformation effect consists of an external suggestion phenomenon in which there is an explicit presentation of the false (suggested) information that is intended to be implanted in the participant’s memory representation. Therefore, in this second paradigm, the aim is to change the original memory representation, whereas in the DRM paradigm, no such aim is pursued; rather, as a result of the presentation of the material, additional information that is related semantically and associatively is activated, and from there, a false memory is derived without modification of a previous representation. Furthermore, both paradigms have clear differences in their translation into the applied field. While the DRM paradigm has been useful in clarifying certain theoretical aspects of generation of false memories but has not generated clear applications, the misinformation paradigm has relevant repercussions in the forensic field, to the extent that it could explain that, in real cases, eyewitnesses change, or add, some details in their accounts of the witnessed event because of a misled question. Moreover, having the same interviewer ask more than one person about the same misleading detail could create a false consensus among eyewitnesses, artificially increasing the perceived accuracy of the detail.

In the next section, we address extreme cases of false memories in the forensic setting which encompass an entire event. One case is about minors who report being victims of sexual abuse after countless interrogations from parents, teachers and other non-experts and experts that are convinced that the sexual abuse has occurred. The other extreme case is about adults with repressed memories of sexual abuse supposedly occurring during their infancy.

## Repressed memories: real or false?

The neurocognitive studies previously described are examples of false memories formed in the laboratory, which have allowed us to clarify the optimal conditions for forming such memories for specific details within an event (i.e., the misinformation paradigm) or for words (in the DRM paradigm). In addition, cognitive studies have delineated which conditions are relevant in forensic cases (for reviews, see Loftus, 1996, 2003, 2005; Diges, 2016).

However, the real cases are far more complex than the paradigms described above. In fact, a good example of that complexity is the phenomenon of memories “rediscovered” in therapy, which occurred in the 1980s and 1990s in the United States. These cases have reappeared with certain force in some European countries during the 21st century (Otgaar et al., 2019, 2022; Lynn et al., 2023).

In the first reported cases of “rediscovered” memories, clients—most of them females—attending psychotherapy sessions were able to remember traumatic episodes, almost always of a sexual nature, which they had supposedly suffered when they were children but not remembered until the psychotherapist had helped them to retrieve the past experience. Apparently, those memories were highly vivid and had a high emotional charge, they were described in detail, and clients blindly believed that these memories were real; they had a very high confidence in them, which, in many cases, led to criminal charges being brought against the alleged perpetrators (almost always fathers or brothers of the women involved). In this forensic context, a question arises as to whether the “recovered” memories are true (in which case they are evidence of the charge) or false (in which case they are not evidence against the defendant). In this situation, is it possible to reliably discriminate between true and false memories of complex episodes?

### The event implantation paradigm and repressed memories

Recovered memories refer to episodes that may have occurred repeatedly many years ago, when the alleged victim was a child (sometimes even a newborn) and which, due to their traumatic nature, have been repressed and stored in the unconscious, i.e., they are repressed memories that are currently exerting a detrimental effect on the person’s physical and/or mental health. For that reason, therapists argue that it is necessary to retrieve these memories and bring them to conscious awareness to be able to deal with them and “heal.” Several techniques have been used to achieve this goal, ranging from direct questioning about the alleged trauma to “memory work” (i.e., triggering or seeking out memories of the alleged abuse), guided imagery, hypnosis, eye movement desensitization and reprocessing (EMDR) therapy, group therapy, and even reincarnation therapy. These techniques are used in repeated sessions over many weeks, even over years, until the client is able to recover the repressed memory.

For their part, experimental psychologists argue that these techniques are very suggestive, and laboratory data have already shown the power of suggestion to modify the recall of details from an episode. Obviously, there is a large difference between making participants believe they saw a false detail pertaining to an event (i.e., the misinformation effect) and making them believe they experienced an entire false episode repeatedly over the years. Thus, Elizbeth Loftus, who was very vocal against the idea that these recovered memories were actually real during the *memory wars* in the 1990s (Otgaar et al., 2019), designed a new experimental procedure, the *event implantation paradigm* (or “lost in the mall” paradigm), which allowed us to test the hypothesis that it is possible to implant memories of childhood events that never occurred in adults.

In contrast to the clinical setting of repressed memories, where there is no control that guarantees that the recall is genuine, the event implantation paradigm allows researchers to control any variable that might prevent them from reaching conclusions. In this case, university students are asked for permission to examine their childhood memories with the collaboration of a relative (usually a parent or sibling). A relative is sent a questionnaire to guarantee the reality of the autobiographical episodes the participant is going to be asked about. The questionnaire includes different types of remarkable events, and the relative must remember whether those events were experienced by his or her son, daughter or sibling. The list of episodes includes situations that could happen to the participant before the age of 6 years and are not extremely traumatic, such as an interaction with a famous public character, a stay in hospital for an injury, the loss of a pet, a prank at school, throwing away the wedding cake at a wedding, or getting lost in a shopping mall.

When the participant’s relative indicates that the participant has experienced such an episode, the relative is asked to provide more information: the approximate age of the participant when the episode occurred, the city in which it occurred, the other people with the participant, etc. From the questionnaire and the information provided by the participant’s relative, a set of experimental episodes are generated. Each participant is asked about a specific set of episodes, which includes three true episodes and a false episode (not remembered as having occurred by the participant’s relative). The set of episodes is presented to the participant as derived from the information provided by his or her relative in the previous questionnaire (Loftus and Pickrell, 1995). The participant is asked to write down what he or she remembers of each episode. The participant is given a general description of the episodes (the same for the invented event and the real events) to serve as a point of departure for remembrance (e.g., “Do you remember that time you got lost in the mall when you were 5 years old?”). Furthermore, once the participant submits his or her responses, he or she is scheduled to be interviewed twice more (approximately one or two weeks apart). The participant is informed that recall could improve the more one thinks about the event, and he or she is even given instructions and homework to do at home (i.e., contextual reinstatement or guided imagination).

In the last interview, between 15% and 25% of participants *remember* the false episode, and under certain experimental conditions, a rate of 37% or even 65% can be reached (Hyman and Pentland, 1996), although the false memory is not usually produced at the first interview (Hyman et al., 1995). The differences in results among studies may be partially due to the induction techniques used, but they could also be due to authors’ conceptualization of false memory because they may differentiate between an entire or clear false memory and a partial false memory. An entire false memory was defined as when a participant admitted that the memory was his or her own and even added data or details that the experimenter had not provided in the initial general description of the episode. A memory was considered partial when the participant accepted it, but he or she did not give any additional detail of what he or she was initially told or described images or conjectures but noted that he or she did not remember it.

Based on this idea, Scoboria et al. (2017) conducted a mega-analysis with 423 memory reports from previously published studies, and they recoded the reports as entire or partial false memories. The overall result showed that 22% of the memory reports were entire or robust false memories and 8.5% were partial false memories, that is, slightly more than 30% of the participants had accepted the suggested event. Even if they obtained a lower false memory rate than they did, with these data, as Loftus and Pickrell (1995) pointed out, “we are only providing ‘proof of existence’ for the phenomenon of false memory formation” (p. 723–724).

It is also important to note that although the suggested episodes had a moderately negative emotional valence,^6^ participants came to believe that they truly experienced these events in childhood over only three interviews.^7^ In psychotherapy sessions that seek repressed memories, most clients initially do not remember the episode of sexual abuse, but a very large number of sessions are devoted to trying to remember it, so it would not be surprising if they eventually succeed; however, this does not guarantee that those memories reflect a real event.

### Repressed memories and forgetting rates

To assess whether memories “rediscovered” in therapy are real memories, one must also take into account the possibility that—as advocated by supporters of the existence of repressed memories and the therapies that recover them—memories of traumatic events may actually have been forgotten for years and can now be recovered in detail without suffering the effects of the passage of time. Based on empirical studies about the effect of trauma on memory, however, Lindsay and Read (1995) pointed out that in real cases, such as witnessing the death of a parent, being hospitalized for physical trauma, or surviving a natural disaster, forgetting is a rare phenomenon.

Additionally, review of what is remembered years after being sexually abused or assaulted as a child does not provide evidence of a different rate of forgetting for these events than for daily experiences. Goodman et al. (2003) found that more than 85% of respondents interviewed approximately 13 years after the assault disclosed in interviews that the abuse had occurred when they were minors. Considering that some of these participants could not remember it, given their young age at the time of the aggression (2 years old) and others did not want to talk about it, repression does not appear to have a strong empirical support.

The same is true for adult victims of real traumatic events, such as Holocaust survivors (Wagenaar and Groeneweg, 1990) or Japanese concentration camp survivors (Merckelbach et al., 2003a,b). However, in these cases, in contrast to the very detailed and vivid memories that are recovered in therapy, memories are of very poor quality, limited to central aspects, and contain distortions as a result of the passage of time. If the memory of patients with posttraumatic stress syndrome is assessed, the problem seems to be the opposite: not only is it not forgotten, but the memory is frequent and invasive and is very distressing.

Additionally, in contrast to understanding of child development and memory, the recovered memories from therapy may be extravagant or even impossible (satanic rituals involving eating babies raw, alien abductions, or reincarnation); however, more importantly, they purportedly were formed at an age affected by infantile amnesia. In general, adults or older children are unable to remember episodes that occurred in their first 4 years of life due to the lack of neurological as well as cognitive and linguistic maturation.

On the other hand, if the memory is repressed by a traumatic event, the victim must understand it as such. However, there are no data that allow us to say that preschoolers sufficiently understand the concept of sexual abuse (for example, if there is a fondling in the genital area) to be able to interpret it in the sense of *trauma*.

Thus, outside these therapies, there is no empirical evidence that traumatic events are repressed, fall out of consciousness, and return to it years later if many recovery attempts are made. Furthermore, the concept of *repression* is scientifically controversial (Lindsay and Read, 2001; Otgaar et al., 2023), although it is widespread and accepted among laypeople (Brewin et al., 2019; Otgaar et al., 2020).

### Repressed memories in the DSM-5: dissociative amnesia

Some authors have drawn attention to the fact that the *Diagnostic and Statistical Manual of Mental Disorders* (American Psychiatric Association, DSM-5 Task Force, 2013) has included dissociative amnesia with a definition that has had “the unfortunate side effect of legitimizing the dubious claim that traumatic memories can be stored yet blocked, only to be retrieved in pristine form years or decades later” (Patihis et al., 2023, p. 381). Furthermore, some of the diagnostic criteria are not very precise (e.g., “ordinary forgetting”); most importantly, the published cases do not meet the criteria of the DSM-5. In their study, Mangiulli et al. (2022) reviewed all cases of dissociative amnesia published in English (in peer-reviewed journals) between 2000 and 2020 (60 articles, 128 cases in total).

According to the DSM-5, the diagnosis of dissociative amnesia requires a loss or impairment of autobiographical memory, usually about an episode (or episodes) of a traumatic or stressful nature. The loss must not be explained by other alternatives, such as malingering, brain damage, ordinary forgetfulness, substance abuse (alcohol or drugs), or other problems, such as posttraumatic stress disorder, traumatic brain injury, or neurocognitive disorders. To rule out these explanations, it is necessary to use specific techniques or tools for each case. For example, malingering tests should be used if we suspect that a person may be pretending (specifically in forensic cases; Peters et al., 2013), retrograde episodic and semantic memory functioning should be assessed (Kopelman, 2000), or data from neuroimaging techniques should be used to exclude altered brain structure (Mangiulli et al., 2022).

The results of the review conducted by Mangiulli et al. (2022) showed that in most cases, only descriptive data on the patient and on the magnitude of the loss in terms of time, type of memory affected (autobiographical, semantic), recovery and use of therapy were reported. However, neuropsychological tests and neuroimaging data were used in less than 50% of the cases. Although in the majority of cases (75%) there were traumatic or stressful episodes prior to the onset of memory loss, many of these cases were potentially confounded by an organic component (e.g., a traffic accident), and sometimes the antecedents of dissociative amnesia were mildly stressful experiences. In fact, virtually none of the published studies presented a rigorous and comprehensive examination of the defining characteristics of dissociative amnesia in the DSM-5 or considered alternative explanations included in the diagnostic manual. In summary, the conclusions of the authors of this critical analysis were that dissociative amnesia in the studies of cases “appeared to be a rather elastic and openly defined construct” (Mangiulli et al., 2022, p. 204) used with diverse types of memory loss, both in the presence and absence of stressors, and without ruling out alternative explanations. It is obvious that the diagnostic criteria are not sufficiently fine, unambiguous or rigorous, so that for each of the problems approached from a clinical point of view it is essential to establish refined and contrasting diagnostic criteria that allow a more precise differential diagnosis and, therefore, a more successful clinical approach.

In summary, published data about cases of dissociative amnesia are scarce and barely meet the criteria established by the DSM-5 for the unequivocal diagnosis of the disorder, even though highly traumatic events are often well remembered (Lynn et al., 2023), and sometimes remembering them is intrusive or pathological (the *sin of persistence*, Schacter, 2022). On the other hand, some therapy practices often lacking credible empirical support (Lynn et al., 2015) have surfaced memories about events that the client did not know he or she had experienced. In almost all cases the repressed memories are objectively and unambiguously corroborated by independent evidence (Bernstein and Loftus, 2009), and they have the richness of detail that would correspond to an adult memory, rather than to the fragmented, naive and simplified interpretations that characterize of childhood memories. From a scientific perspective, we have no technique (cognitive or neural) that allows us to distinguish true memories from false memories (Bernstein and Loftus, 2009), but this does not mean that cases cannot be analyzed from a forensic psychology point of view.

Like other evidence, recovered memory can be explained by a number of alternative hypotheses. Dissociative amnesia is one such explanation, and we have seen that it presents serious diagnostic problems in the literature. The suggestive techniques employed by many therapists seeking such memories provide another explanation to consider. As seen in the misleading and memory implantation studies, certain suggestive techniques, such as guided imagination, peer pressure (Herndon et al., 2014), repeated questions, hypnosis (Mazzoni and Memon, 2003; Mazzoni and Lynn, 2007) and lateral movements (Houben et al., 2018) have been used to create a false memory in very few sessions (usually three), which the participant comes to believe. These same techniques, together with pseudoscientific practices such as neurolinguistic programming, sensorimotor psychotherapy, alien abduction therapy or primal therapy (Lynn et al., 2023), applied in a much larger number of sessions, could also lead to false memories (Lilienfeld, 2007). If these techniques have been used and the client has recovered a previously unknown memory, it cannot be ruled out that this memory is the result of suggestive influences rather than a genuine recollection of a truly vivid episode.

This is exactly the same problem observed with the statements of children in alleged cases of childhood sexual abuse (CSA): the inability to reject the suggestion hypothesis when a minor discloses alleged abuse.

### Statements of children in alleged cases of CSA

In European and North American criminal courts, when the alleged victim is a minor, the case is often related to childhood sexual abuse (CSA). In these cases, there is usually no physical evidence or witnesses to corroborate the child’s allegations, so the child’s statement is the only prosecution evidence.

In this situation, it is important to question the child in a nonsuggestive manner so as not to contaminate the evidence, i.e., the child’s statement. However, it is possible that suggestive sources of information may influence the child’s statements even before the formal report of CSA, at is likely that parents or others close to the child will have asked the child if there is a suspicion that he or she may have been sexually abused. The questions asked by parents, teachers, and even physicians are often carelessly repetitive and usually have high rates of “off the record” suggestions, especially if the child is very young (e.g., preschool age); thus, repeated questions from different sources often contaminate the child’s statement about sexual abuse (Korkman et al., 2014). Therefore, before the police investigation begins, the evidence could have a high degree of contamination, to which even more suggestive influences can be added if polices, judges and psychologists do not take extreme care in questioning the child about the alleged abuse.

Today, we know that the factors that increase the misinformation effect are the same factors that increase the contamination of testimony or the possibility of creating a testimony. The person who asks (parent, teacher, physician, police, judge) has high credibility (Dodd and Bradshaw, 1980; Ceci et al., 1987). Furthermore, he or she can ask many questions to the minor in a repetitive and suggestive way (Bruck et al., 1995) until he or she obtains an answer that confirms his or her expectations. The suggestive questions are asked both in the same session or interview and over several sessions (Poole and White, 1991), and the child’s answer is positively reinforced when it fits with the expectations of those who ask the questions, while it is rejected when it does not fit with those expectations (Garven et al., 2000). On some occasions, the child is pressed (“Others have told me that…,” Garven et al., 1998), and even it is encouraged to speculate or simulate actions with dolls.^8^ The result of these types of questions is the contamination of the statement if the episode was real; however, sometimes a false memory can be formed when the episode was not real, but suggestive questions were asked led to the assumption that the abuse had occurred (Garven et al., 2000).

From a forensic perspective, a problem arises when a false memory is not very different from a true memory because the information suggested through the questions asked leads to embellishment of the genuine memory (Principe and Ceci, 2002; Principe et al., 2006; Peláez Devesa et al., 2019). Currently, given the contributions of cognitive psychology and experimental studies on suggestion, we are able to assess the possibility that a memory may be embellished or may even have arisen ex novo from poorly formulated questions. On the other hand, from a neural point of view, it is not possible to indisputably determine whether a memory is true or false.

As in the case of repressed false memories recovered in therapy, experimental data allow us to evaluate the possibility that a given memory refers to a real event, provided we can rule out the alternative hypotheses of suggestion and lying. Obviously, the key question is the ability to conduct adequate hypothesis testing. Cognitively, we have the necessary tools, although it is not a foolproof process for all cases. At the neural level, we currently lack the tool for assessing complete episodes supposedly experienced in the past. Therefore, to rigorously analyze the statements—i.e., the prosecution evidence—of the alleged victims of sexual abuse, we must continue to rely on the contributions of cognitive psychology to the functioning of memory, language and other sociocognitive processes in the development of the minors involved.

## Conclusion

After nearly half a century of research on the formation of false memories, traditional and recent approaches reveal that it is impossible to completely discriminate between true and false memories, both for the person experiencing those memories and for the person who must judge them.

None of the myriad and varied measures employed—accuracy, reaction times, confidence estimates, phenomenological judgments, ERP activity, or brain activity recorded by diverse and sophisticated neuroimaging techniques—have revealed a genuine and unique signature for each type of memory (true and false). Thus, a memory report or statement is an open problem whose resolution involves a rigorous and exhaustive process of hypothesis testing to identify the hypothesis (or hypotheses) that best explain the current content and quality of the memory report or statement. This requires a careful analysis of the encoding conditions of the episode, the duration of the retention interval and what occurred during that time span, and the conditions of retrieval of the episode.

With the fruitful cognitive approach to the study of true and false memories, it has been possible to establish the most relevant factors to be considered when analyzing a statement in the forensic setting. On the other hand, despite intriguing contributions, research on brain activity related to false memories must advance before making the leap to the forensic field, should this be possible in the future.

In summary, currently, for criminal cases in which the only prosecution evidence comes from the memory of a witness or victim, the most effective psychological tool at our disposal is the rigorous testing of hypotheses based on scientific knowledge about the functioning of memory and other interrelated sociocognitive processes, as well as the factors that affect them. In this sense, future lines of research should aim to delve deeper into these processes and factors, especially in more complex cases where a wide range of factors may be involved. For example, there are cases that have a large social and media impact, in which there are factors that carry a lot of weight and are not as well explored or delineated. Social factors are particularly important in alleged sexual abuse with multiple victims-in formal or non-formal educational contexts-, where the exchange or exposure of information in networks requires special attention since it is an important source of contamination of the testimonies. Another research topic that needs more attention and that is relevant to the forensic field is the functioning of memory and the vulnerability to suggestion of people with functional diversity and disabilities. There is scarce research in this field and there is an urgent need to dedicate recourses to it. Furthermore, in this topic is quite more limited the contribution of neuroscience because the added difficulty to get reliable data with the ERP or neuroimaging methodologies in these populations. Last, but not less important, the processes of hypothesis testing must be well defined to the professionals who are responsible for the analysis of eyewitnesses’ statements to reach decisions based on a rigorous work that guarantees the minimum margin of error. This is also an interesting topic to address because the process of testing the hypotheses that explain the quality and content of a statement is where the most errors are detected in the expert reports carried out by professionals.

Despite the differences in the approach to the study of false memories at the two levels of analysis that we have examined in this paper, cognition and brain activity in essence are telling the same story: on a case-by-case it is almost impossible to reliably discriminate between true and false memories, although cognitive research at least gives us the possibility to make a rigor analysis of each case.

## Author contributions

NP-M: Writing – review & editing, Writing – original draft, Conceptualization. MD: Writing – original draft, Conceptualization.

## Footnotes

^1^For the implantation of low plausibility events (e.g., receiving an enema in childhood) and extremely low plausibility events (e.g., seeing a ghost), see Sharman and Scoboria (2009, 2011).

^2^Participants were divided into groups of “good” and “poor” performers based on their ability to discriminate between studied words and critical lures. For that, A median split was used to separate participants into good and poor performers based on the discrimination of studied targets from critical lures.

^3^TDCS is believed to directly manipulate ongoing cortical activity by passing a direct current stimulation though the scalp from a positive electrode (anode) to a negative electrode (cathode) (for review, see Nitsche et al., 2008). The opposing polarities of each electrode are thought to result in opposing polarization of the neuronal membrane such that anodal stimulation increases cortical excitability, whereas cathodal stimulation decreases it (Nitsche et al., 2005; Reato et al., 2010).

^4^This is a relative new technique with a high temporal resolution and safety that allows the study of memory process in the cortex. Light emitting diodes (LEDs) placed on the scalp emit two wavelengths of infrared light that is absorbed by hemoglobin in the blood enabling the quantification of the blood oxygenation level.

^5^Note that a factor partly responsible for the inconsistent patterns of results obtained in neuroimaging studies could be the size of samples used. This question goes beyond the objectives of the present work, but for a rigorous analysis of the effects of sample size on the results obtained and the different statistical type of errors involved in neuroimaging research, see Marek et al. (2022).

^6^Some studies, such as Herndon et al. (2014), have established that a very painful medical procedure involving genital contact (voiding cysto-urethrogram or VCUG) is a traumatic event, but under certain experimental conditions, 75% of participants were able to form false memories of the episode.

^7^The design of these studies, with adults or children as participants, deeply consider ethical issues with regard the risks of each situation. In the first publications using this paradigm, methodology was pretested to confirm that nobody felt discomfort or stress, and during the interviews participants were scrutinized for signs of distress (see Ceci et al., 1998). Also, Murphy et al. (2023) asked participants and informants about their experience in a replication of the study of Loftus and Pickrell (1995), and they found that participants and informants felt very positive and considered the deceptive methods acceptable. It is now common practice to pre-register study procedures and materials and/or obtain approval from the ethics committee of the institution with which the researchers are affiliated to ensure that the study complies with the research ethics code.

^8^A very complete review about conditions that enhance the misinformation effect on children can be found in Ceci and Bruck (1995, 2006) and Bruck and Ceci (1999).

## References

[ref1] Abe N. (2012). Neuroimaging studies of false memory: a selective review. Psychologia 55, 131–145. doi: 10.2117/psysoc.2012.131

[ref2] Abe N. Jiro O. Maki S. Hiroshi S. Tetsuya M. Etsuro M.. (2008). Neural correlates of true memory, false memory, and deception. Cereb. Cortex 18, 2811–2819. doi: 10.1093/cercor/bhn037, PMID: 18372290 PMC2583150

[ref3] Aguirre G. K. Feinberg F. T. Farah M. J. (2003). “Functional imaging in behavioral neurology and cognitive neuropsychology” in Behavioral neurology and cognitive neuropsychology. eds. Feinberg T. E. Farah M. J. (New York: McGraw-Hill), 85–96.

[ref4] Allan K. Wilding L. Rugg M. D. (1998). Electrophysiological evidence for dissociable processes contributing to recollection. Acta Psychol. 98, 231–252. doi: 10.1016/S0001-6918(97)00044-9, PMID: 9621832

[ref5] Ambach W. Bursch S. Stark R. Vaitl D. (2010). A concealed information test with multimodal measurement. Int. J. Psychophysiol. 75, 258–267. doi: 10.1016/j.ijpsycho.2009.12.007, PMID: 20026133

[ref6] American Psychiatric Association, DSM-5 Task Force (2013). Diagnostic and statistical manual of mental disorders: DSM-5™ (5th). American Psychiatric Publishing, Inc. Washington, DC

[ref7] Arndt J. (2012). “False recollection: empirical findings and their theoretical implications” in Psychology of learning and motivation. ed. Ross B. H. (Cambridge, MA: Academic Press), 81–124.

[ref8] Baym C. L. Gonsalves B. D. (2010). Comparison of neural activity that leads to true memories, false memories, and forgetting: an fMRI study of the misinformation effect. Cogn. Affect. Behav. Neurosci. 10, 339–348. doi: 10.3758/cabn.10.3.339, PMID: 20805535

[ref9] Beato M. S. Boldini A. Cadavid S. (2012). False memory and level of processing effect: an event-related potential study. Neuroreport 23, 804–808. doi: 10.1097/WNR.0b013e32835734de, PMID: 22811058

[ref10] Bernstein D. M. Loftus E. F. (2009). How to tell if a particular memory is true or false. Perspect. Psychol. Sci. 4, 370–374. doi: 10.1111/j.1745-6924.2009.01140.x 26158984

[ref11] Boggio P. S. Fregni F. Valasek C. Ellwood S. Chi R. Gallate J.. (2009). Temporal lobe cortical electrical stimulation during the encoding and retrieval phase reduces false memories. PLoS One 4:e4959. doi: 10.1371/journal.pone.0004959, PMID: 19319182 PMC2655647

[ref12] Boldini A. Beato M. S. Cadavid S. (2013). Modality-match effect in false recognition: an event-related potential study. Neuroreport 24, 108–113. doi: 10.1097/WNR.0b013e32835c93e3 23370492

[ref13] Brainerd C. J. Reyna V. F. (1998). When things that were never experienced are easier to "remember" than things that were. Psychol. Sci. 9, 484–489. doi: 10.1111/1467-9280.00089

[ref14] Brainerd C. J. Reyna V. F. (2002). Fuzzy-trace theory and false memory. Curr. Dir. Psychol. Sci. 11, 164–169. doi: 10.1111/1467-8721.00192

[ref15] Brewin C. R. Li H. Ntarantana V. Unsworth C. McNeilis J. (2019). Is the public understanding of memory prone to widespread “myths”? J. Exp. Psychol. Gen. 148, 2245–2257. doi: 10.1037/xge0000610, PMID: 31033323

[ref16] Bruck M. Ceci S. J. (1999). The suggestibility of children’s memory. Annu. Rev. Psychol. 50, 419–439. doi: 10.1146/annurev.psych.50.1.419 10074684

[ref17] Bruck M. Ceci S. J. Francoeur E. Barr R. (1995). “I hardly cried when I got my shot!” influencing children's reports about a visit to their pediatrician. Child Dev. 66, 193–208. doi: 10.1111/j.1467-8624.1995.tb00865.x, PMID: 7497825

[ref18] Cabeza R. Rao S. M. Wagner A. D. Mayer A. R. Schacter D. L. (2001). Can medial temporal lobe regions distinguish true from false? An event-related functional MRI study of veridical and illusory recognition memory. Proc. Natl. Acad. Sci. USA 98, 4805–4810. doi: 10.1073/pnas.081082698, PMID: 11287664 PMC31915

[ref19] Cadavid S. Beato M. S. (2016). Memory distortion and its avoidance: an event-related potentials study on false recognition and correct rejection. PLoS One 11:e0164024. doi: 10.1371/journal.pone.0164024, PMID: 27711125 PMC5053520

[ref20] Ceci S. J. Bruck M. (1995). Jeopardy in the courtroom: a scientific analysis of children's testimony. American Psychological Association. Washington, DC

[ref21] Ceci S. J. Bruck M. (2006). Children’s suggestibility: characteristic and mechanisms. Adv. Child Dev. Behav. 34, 247–281. doi: 10.1016/S0065-2407(06)80009-1 17120807

[ref22] Ceci S. J. Bruck M. Loftus E. F. (1998). On the ethics of memory implantation research. Appl. Cogn. Psychol. 12, 230–240. doi: 10.1002/(SICI)1099-0720(199806)12:3<230::AID-ACP526>3.0.CO;2-1

[ref23] Ceci S. J. Ross D. F. Toglia M. P. (1987). Suggestibility of children's memory: Psycholegal implications. J. Exp. Psychol. Gen. 116, 38–49. doi: 10.1037/0096-3445.116.1.38

[ref24] Chadwick M. J. Anjum R. S. Kumaran D. Schacter D. L. Spiers H. J. Hassabis D. (2016). Semantic representations in the temporal pole predict false memories. Proc. Natl. Acad. Sci. 113, 10180–10185. doi: 10.1073/pnas.1610686113 27551087 PMC5018755

[ref25] Chen J. C. Li W. Westerberg C. E. Tzeng O. J. (2008). Test-item sequence affects false memory formation: an event-related potential study. Neurosci. Lett. 431, 51–56. doi: 10.1016/j.neulet.2007.11.020, PMID: 18155357

[ref26] Coburn P. I. Dogra K. K. Rai I. K. Bernstein D. M. (2021). The trajectory of targets and critical lures in the Deese/Roediger–McDermott paradigm: a systematic review. Front. Psychol. 12, 1–15. doi: 10.3389/fpsyg.2021.718818, PMID: 34925128 PMC8677658

[ref27] Cruse D. Wilding E. L. (2009). Prefrontal cortex contributions to episodic retrieval monitoring and evaluation. Neuropsychologia 47, 2779–2789. doi: 10.1016/j.neuropsychologia.2009.06.003 19523968

[ref28] Curran T. (2000). Brain potentials of recollection and familiarity. Mem. Cogn. 28, 923–938. doi: 10.3758/BF03209340 11105518

[ref29] Curran T. Cleary A. M. (2003). Using ERPs to dissociate recollection from familiarity in picture recognition. Cogn. Brain Res. 15, 191–205. doi: 10.1016/S0926-6410(02)00192-1, PMID: 12429370

[ref30] Curran T. Dien J. (2003). Differentiating amodal familiarity from modality-specific memory processes: an ERP study. Psychophysiology 40, 979–988. doi: 10.1111/1469-8986.00116, PMID: 14986851 PMC1413574

[ref31] Curran T. Hancock J. (2007). The FN400 indexes familiarity-based recognition of faces. NeuroImage 36, 464–471. doi: 10.1016/j.neuroimage.2006.12.016, PMID: 17258471 PMC1948028

[ref32] Curran T. Schacter D. L. Johnson M. K. Spinks R. (2001). Brain potentials reflect behavioral differences in true and false recognition. J. Cogn. Neurosci. 13, 201–216. doi: 10.1162/089892901564261, PMID: 11244546

[ref33] Deese J. (1959). On the prediction of occurrence of particular verbal intrusions in immediate recall. J. Exp. Psychol. 58, 17–22. doi: 10.1037/h0046671, PMID: 13664879

[ref34] Dennis N. A. Bowman C. R. Turney I. C. (2015). “Functional neuroimaging of false memories” in The Wiley handbook on the cognitive neuroscience of memory. eds. Addis D. R. Barense M. Duarte A.. 1st ed (Hoboken, NJ: Jonh Wiley & Sons, Ltd.), 150–171.

[ref35] Dennis N. A. Chamberlain J. D. Carpenter C. M. (2023). “False memories: what neuroimaging tells us about how we Mis-remember the past” in The SAGE handbook of cognitive and systems neuroscience. Cognitive systems, development and applications. eds. Boyle G. J. Northoff G. Barbey A. K. Fregni F. Jahanshani M. Pascual-Leone A.. (Thousand Oaks, CA: SAGE Publications), 91–73.

[ref36] Díez E. Gómez-Ariza C. J. Díez-Álamo A. M. Alonso M. A. Fernandez A. (2017). The processing of semantic relatedness in the brain: evidence from associative and categorical false recognition effects following transcranial direct current stimulation of the left anterior temporal lobe. Cortex 93, 133–145. doi: 10.1016/j.cortex.2017.05.004, PMID: 28647325

[ref37] Diges M. (2016). Testigos, sospechosos, y recuerdos falsos. Estudios de Psicología Forense. Madrid: Editorial Trotta.

[ref38] Dodd D. H. Bradshaw J. M. (1980). Leading questions and memory: pragmatic constraints. J. Verbal Learn. Verbal Behav. 19, 695–704. doi: 10.1016/S0022-5371(80)90379-5

[ref39] Düzel E. Yonelinas A. P. Mangun G. R. Heinze H. Tulving E. (1997). Event-related brain potential correlates of two states of conscious awareness in memory. Proc. Natl. Acad. Sci. USA 94, 5973–5978. doi: 10.1073/pnas.94.11.5973, PMID: 9159185 PMC20891

[ref40] Edelson M. Sharot T. Dolan R. J. Dudai Y. (2011). Following the crowd: brain substrates of long-term memory conformity. Sciences 333, 108–111. doi: 10.1126/science.1203557, PMID: 21719681 PMC3284232

[ref41] Favre G. Horat S. K. Herrmann F. R. Gothuey I. Merlo M. Missonnier P. (2020). Neurophysiological signature of memory performance during the DRM task. Int. J. Cogn. Ther. 3, 1–10. doi: 10.23937/2690-3172/1710007

[ref42] Finnigan S. Humphreys M. S. Dennis S. Geffen G. (2002). ERP ‘old/new’ effects: memory strength and decisional factor(s). Neuropsychologia 40, 2288–2304. doi: 10.1016/S0028-3932(02)00113-6 12417459

[ref43] Friedman D. Johnson R. Jr. (2000). Event-related potential (ERP) studies of memory encoding and retrieval: a selective review. Microsc. Res. Tech. 51, 6–28. doi: 10.1002/1097-0029(20001001)51:1, PMID: 11002349

[ref44] Gallate J. Chi R. Ellwood S. Snyder A. (2009). Reducing false memories by magnetic pulse stimulation. Neurosci. Lett. 449, 151–154. doi: 10.1016/j.neulet.2008.11.021, PMID: 19022348

[ref45] Gallo D. A. (2010). False memories and fantastic beliefs: 15 years of the DRM illusion. Mem. Cogn. 38, 833–848. doi: 10.3758/MC.38.7.833, PMID: 20921097

[ref46] Gamer M. Berti S. (2012). P300 amplitudes in the concealed information test are less affected by depth of processing than electrodermal responses. Front. Hum. Neurosci. 6, 1–10. doi: 10.3389/fnhum.2012.00308, PMID: 23162454 PMC3498630

[ref47] Ganis G. Schendan H. E. (2013). Concealed semantic and episodic autobiographical memory electrified. Hum. Neurosci. 6, 1–21. doi: 10.3389/fnhum.2012.00354 PMC3553422 23355816

[ref48] Garven S. Wood J. M. Malpass R. S. (2000). Allegations of wrongdoing: the effects of reinforcement on children's mundane and fantastic claims. J. Appl. Psychol. 85, 38–49. doi: 10.1037/0021-9010.85.1.38, PMID: 10740955

[ref49] Garven S. Wood J. M. Malpass R. S. Shaw J. S. (1998). More than suggestion. The effect of interviewing techniques from the McMartin preschool case. J. Appl. Psychol. 83, 347–359. doi: 10.1037/0021-9010.83.3.347 9648524

[ref50] Gatti D. Vecchi T. Mazzoni G. (2021). Cerebellum and semantic memory: a TMS study using the DRM paradigm. Cortex 135, 78–91. doi: 10.1016/j.cortex.2020.11.017 33360762

[ref51] Gonsalves B. D. Kahn I. Curran T. Norman K. A. Wagner A. D. (2005). Memory strength and repetition suppression: multimodal imaging of medial temporal cortical contributions to recognition. Neuron 47, 751–761. doi: 10.1016/j.neuron.2005.07.013, PMID: 16129403

[ref52] Goodman G. S. Ghetti S. Quas J. A. Edelstein R. S. Alexander K. W. Redlich A. D.. (2003). A prospective study of memory for child sexual abuse: new findings relevant to the repressed-memory controversy. Psychol. Sci. 14, 113–118. doi: 10.1111/1467-9280.01428, PMID: 12661671

[ref53] Gordon A. Brooks J. C. Quadflieg S. Ecker U. K. Lewandowsky S. (2017). Exploring the neural substrates of misinformation processing. Neuropsychologia 106, 216–224. doi: 10.1016/j.neuropsychologia.2017.10.003, PMID: 28987910

[ref54] Hayama H. R. Johnson J. D. Rugg M. D. (2008). The relationship between the right frontal old/new ERP effect and post-retrieval monitoring: specific or non-specific? Neuropsychologia 46, 1211–1223. doi: 10.1016/j.neuropsychologia.2007.11.021, PMID: 18234241 PMC2441597

[ref55] Henson R. (2005). What can functional neuroimaging tell the experimental psychologist? Q J. Exp. Psychol. A 58, 193–233. doi: 10.1080/02724980443000502, PMID: 15903115

[ref56] Herndon P. Myers B. Mitchell K. Kehn A. Henry S. (2014). False memories for highly aversive early childhood events: effects of guided imagery and group influence. Psychol. Conscious. Theory Res. Pract. 1, 20–31. doi: 10.1037/cns0000011

[ref57] Houben S. T. Otgaar H. Roelofs J. Merckelbach H. (2018). Lateral eye movements increase false memory rates. Clin. Psychol. Sci. 6, 610–616. doi: 10.1177/2167702618757658, PMID: 30101041 PMC6056909

[ref58] Hyman I. E. Billings J. (1998). Individual differences and the creation of false childhood memories. Memory 6, 1–20. doi: 10.1080/741941598, PMID: 9640430

[ref59] Hyman I. E. Husband T. H. Billings F. J. (1995). False memories of childhood experiences. Appl. Cogn. Psychol. 9, 181–197. doi: 10.1002/acp.2350090302

[ref60] Hyman I. E. Pentland J. (1996). The role of mental imagery in the creation of false childhood memories. J. Mem. Lang. 35, 101–117. doi: 10.1006/jmla.1996.0006

[ref61] Johnson R. (1995). “Event-related potential insights into the neurobiology of memory systems” in Handbook of neuropsychology. eds. Baron J. C. Grafman J. (Amsterdam: Elsevier), 135–163.

[ref62] Johnson M. K. Hashtroudi S. Lindsay D. S. (1993). Source monitoring. Psychol. Bull. 114, 3–28. doi: 10.1037/0033-2909.114.1.3 8346328

[ref63] Johnson M. K. Kounios J. Nolde S. F. (1997). Electrophysiological brain activity and memory source monitoring. Neuroreport 8, 1317–1320. doi: 10.1097/00001756-199703240-00051, PMID: 9175136

[ref64] Johnson M. K. Raye C. L. Mitchell K. J. (2012). “The cognitive neuroscience of true and false memories” in True and false recovered memories: Toward a reconciliation of the debate, Nebraska symposium on motivation. ed. Belli R. F. (Berlin: Springer Science+Business Media, LLC2012), 15–52. 10.1007/978-1-4614-1195-6_2 22303763

[ref65] Jou J. Flores S. (2013). How are false memories distinguishable from true memories in the Deese–Roediger–McDermott paradigm? A review of the findings. Psychol. Res. 77, 671–686. doi: 10.1007/s00426-012-0472-6, PMID: 23266577

[ref66] Kiat J. Belli R. (2017). An exploratory high-density EEG investigation of the misinformation effect: attentional and Recollective differences between true and false perceptual memories. Neurobiol. Learn. Mem. 141, 199–208. doi: 10.1016/j.nlm.2017.04.007, PMID: 28442391

[ref67] Kim H. Cabeza R. (2007a). Differential contributions of prefrontal, medial temporal, and sensory–perceptual regions to true and false memory formation. Cereb. Cortex 17, 2143–2150. doi: 10.1093/cercor/bhl122, PMID: 17110592

[ref68] Kim H. Cabeza R. (2007b). Trusting our memories: dissociating the neural correlates of confidence in veridical versus illusory memories. J. Neurosci. 27, 12190–12197. doi: 10.1523/JNEUROSCI.3408-07.2007, PMID: 17989285 PMC6673246

[ref69] Kopelman M. D. (2000). Focal retrograde amnesia and the attribution of causality: an exceptionally critical view. Cogn. Neuropsychol. 17, 585–621. doi: 10.1080/026432900750002172, PMID: 20945196

[ref70] Korkman J. Juusola A. Santtila P. (2014). Who made the disclosure? Recorded discussions between children and caretakers suspecting child abuse. Psychol. Crime Law 20, 994–1004. doi: 10.1080/1068316X.2014.902455

[ref71] Kuo T. Van Petten C. (2006). Prefrontal engagement during source memory retrieval depends on the prior encoding task. J. Cogn. Neurosci. 18, 1133–1146. doi: 10.1162/jocn.2006.18.7.1133 16839287 PMC2507728

[ref72] Kuo T. Y. Van Petten C. (2008). Perceptual difficulty in source memory encoding and retrieval: prefrontal versus parietal electrical brain activity. Neuropsychologia 46, 2243–2257. doi: 10.1016/j.neuropsychologia.2008.02.018, PMID: 18402989 PMC2409272

[ref73] Kurkela K. A. Dennis N. A. (2016). Event-related fMRI studies of false memory: an activation likelihood estimation meta-analysis. Neuropsychologia 81, 149–167. doi: 10.1016/j.neuropsychologia.2015.12.006, PMID: 26683385

[ref74] Lahnakoski J. M. Jääskeläinen I. P. Sams M. Nummenmaa L. (2017). Neural mechanisms for integrating consecutive and interleaved natural events. Hum. Brain Mapp. 38, 3360–3376. doi: 10.1002/hbm.23591, PMID: 28379608 PMC6866821

[ref75] Lewandowsky S. Ecker U. K. H. Seifert C. M. Schwarz N. Cook J. (2012). Misinformation and its correction: continued influence and successful debiasing. Psychol. Sci. Public Interest 13, 106–131. doi: 10.1177/1529100612451018 26173286

[ref76] Lilienfeld S. O. (2007). Psychological treatments that cause harm. Perspect. Psychol. Sci. 2, 53–70. doi: 10.1111/j.1745-6916.2007.00029.x 26151919

[ref77] Lindsay D. S. Johnson M. K. (1989). The eyewitness suggestibility effect and memory for source. Mem. Cogn. 17, 349–358. doi: 10.3758/BF03198473, PMID: 2725271

[ref78] Lindsay D. S. Read J. D. (1994). Psychotherapy and memories of childhood sexual abuse: a cognitive perspective. Appl. Cogn. Psychol. 8, 281–338. doi: 10.1002/acp.2350080403

[ref79] Lindsay D. S. Read J. D. (1995). "memory work" and recovered memories of childhood sexual abuse: scientific evidence and public, professional, and personal issues. Psychol. Public Policy Law 1, 846–908. doi: 10.1037/1076-8971.1.4.846

[ref80] Lindsay D. S. Read J. D. (2001). “The recovered memories controversy: where do we go from here?” in Recovered memories: Seeking the middle ground. eds. Davies G. M. Dalgleish T. (Chichester, UK: Wiley), 71–93.

[ref81] Loftus E. F. (1975). Leading questions and the eyewitness report. Cogn. Psychol. 7, 560–572. doi: 10.1016/0010-0285(75)90023-7

[ref82] Loftus E. F. (1996). Eyewitness testimony. Harvard University Press, Cambridge, MA.

[ref83] Loftus E. F. (2003). Our changeable memories: legal and practical implications. Nat. Rev. Neurosci. 4, 231–234. doi: 10.1038/nrn1054, PMID: 12612635

[ref84] Loftus E. F. (2005). Planting misinformation in the human mind: a 30-year investigation of the malleability of memory. Learn. Mem. 12, 361–366. doi: 10.1101/lm.94705 16027179

[ref85] Loftus E. F. Pickrell J. E. (1995). The formation of false memories. Psychiatr. Ann. 25, 720–725. doi: 10.3928/0048-5713-19951201-07

[ref86] Lynn S. J. Evans J. Laurence J. R. Lilienfeld S. O. (2015). What do people believe about memory? Implications for the science and pseudoscience of clinical practice. Can. J. Psychiatry 60, 541–547. doi: 10.1177/070674371506001204, PMID: 26720822 PMC4679162

[ref87] Lynn S. J. McNally R. J. Loftus E. F. (2023). The memory wars then and now: the contributions of Scott O. Lilienfeld. Clin. Psychol. Sci. 11, 725–743. doi: 10.1177/21677026221133034

[ref88] Mangiulli I. Otgaar H. Jelicic M. Merckelbach H. (2022). A critical review of case studies on dissociative amnesia. Clin. Psychol. Sci. 10, 191–211. doi: 10.1177/21677026211018194

[ref89] Marek S. Tervo-Clemmens B. Calabro F. J. Montez D. F. Kay B. P. Hatoum A. S.. (2022). Reproducible brain-wide association studies require thousands of individuals. Nature 603, 654–660. doi: 10.1038/s41586-022-04492-9, PMID: 35296861 PMC8991999

[ref90] Matsuda I. Nittono H. Ogawa T. (2011). Event-related potentials increase the discrimination performance of the autonomic-based concealed information test. Psychophysiology 48, 1701–1710. doi: 10.1111/j.1469-8986.2011.01266.x, PMID: 21806637

[ref91] Mazzoni G. Lynn S. J. (2007). “Using hypnosis in eyewitness memory: past and current issues” in The handbook of eyewitness psychology, Vol. 1. Memory for events. eds. Toglia M. P. Read J. D. Ross D. F. Lindsay R. C. L. (Mahwah: Lawrence Erlbaum Associates Publishers), 321–338.

[ref92] Mazzoni G. Memon A. (2003). Imagination can create false autobiographical memories. Psychol. Sci. 14, 186–188. doi: 10.1046/j.1432-1327.1999.00020.x, PMID: 12661683

[ref93] McCloskey M. Zaragoza M. (1985). Misleading postevent information and memory for events: arguments and evidence against memory impairment hypothesis. J. Exp. Psychol. Gen. 114, 1–16. doi: 10.1037/0096-3445.114.1.1, PMID: 3156942

[ref94] McDermott K. B. Gilmore A. W. Nelson S. M. Watson J. M. Ojemann J. G. (2017). The parietal memory network actives similarity for true and associative false recognition elicited via the DRM procedure. Cortex 87, 96–107. doi: 10.1016/j.cortex.2016.09.008, PMID: 27745847

[ref95] Mecklinger A. (2000). Interfacing mind and brain: a neurocognitive model of recognition memory. Psychophysiology 37, 565–582. doi: 10.1111/1469-8986.3750565, PMID: 11037034

[ref96] Mecklinger A. Meinshausen R. (1998). Recognition memory for object form and object location: an event-related potential study. Mem. Cogn. 26, 1068–1088. doi: 10.3758/BF03201184 9796237

[ref97] Meek S. W. Phillips M. C. Boswell C. P. Vendemia J. M. (2013). Deception and the misinformation effect: an event-related potential study. Int. J. Psychophysiol. 87, 81–87. doi: 10.1016/j.ijpsycho.2012.11.004, PMID: 23183315

[ref98] Meixner J. B. Rosenfeld J. P. (2014). Detecting knowledge of incidentally acquired, real-world memories using a P300-based concealed-information test. Psychol. Sci. 25, 1994–2005. doi: 10.1177/0956797614547278, PMID: 25231899

[ref99] Merckelbach H. Dekkers T. Wessel I. Roefs A. (2003a). Amnesia, flashbacks, nightmares, and dissociation in aging concentration camp survivors. Behav. Res. Ther. 41, 351–360. doi: 10.1016/S0005-7967(02)00019-0 12600404

[ref100] Merckelbach H. Dekkers T. Wessel I. Roefs A. J. (2003b). Dissociative symptoms and amnesia in Dutch concentration camp survivors. Compr. Psychiatry 44, 65–69. doi: 10.1053/comp.2003.50011, PMID: 12524638

[ref101] Morcom A. M. (2015). Resisting false recognition: an ERP study of lure discrimination. Brain Res. 1624, 336–348. doi: 10.1016/j.brainres.2015.07.049 26256253

[ref102] Murphy G. Maher J. Ballantyne L. Barrett E. Cowman C. S. Dawson C. A.. (2023). How do participants feel about the ethics of rich false memory studies? Memory 31, 474–481. doi: 10.1080/09658211.2023.2170417 36689341

[ref103] Nitsche M. A. Cohen L. G. Wassermann E. M. Priori A. Lang N. Antal A.. (2008). Transcranial direct current stimulation: state of the art 2008. Brain Stimul. 1, 206–223. doi: 10.1016/j.brs.2008.06.004, PMID: 20633386

[ref104] Nitsche M. A. Seeber A. Frommann K. Klein C. C. Rochford C. Nitsche M. S.. (2005). Modulating parameters of excitability during and after transcranial direct current stimulation of the human motor cortex. J. Physiol. 568, 291–303. doi: 10.1113/jphysiol.2005.092429, PMID: 16002441 PMC1474757

[ref105] Okado Y. Stark C. E. L. (2005). Neural activity during encoding predicts false memories created by misinformation. Learn. Mem. 12, 3–11. doi: 10.1101/lm.87605, PMID: 15687227 PMC548489

[ref106] Otgaar H. Curci A. Mangiulli I. Battista F. Rizzotti E. Sartori G. (2022). A court ruled case on therapy-induced false memories. J. Forensic Sci. 67, 2122–2129. doi: 10.1111/1556-4029.15073, PMID: 35652501 PMC9544012

[ref107] Otgaar H. Dodier O. Garry M. Howe M. L. Loftus E. F. Lynn S. J.. (2023). Oversimplifications and misrepresentations in the repressed memory debate: a reply to Ross. J. Child Sex. Abus. 32, 116–126. doi: 10.1080/10538712.2022.2133043, PMID: 36229991

[ref108] Otgaar H. Howe M. L. Patihis L. Merckelbach H. Lynn S. J. Lilienfeld S. O.. (2019). The return of the repressed: the persistent and problematic claims of long-forgotten trauma. Perspect. Psychol. Sci. 14, 1072–1095. doi: 10.1177/1745691619862306, PMID: 31584864 PMC6826861

[ref109] Otgaar H. Wang J. Howe M. L. Lilienfeld S. O. Loftus E. F. Lynn S. J.. (2020). Belief in unconscious repressed memory is widespread: a comment on Brewin, Li, Ntarantana, Unsworth, and McNeilis (2019). J. Exp. Psychol. Gen. 149, 1996–2000. doi: 10.1037/xge0000721, PMID: 33017163

[ref110] Paller K. A. Kutas M. (1992). Brain potentials during memory retrieval provide neurophysiological support for the distinction between conscious recollection and priming. J. Cogn. Neurosci. 4, 375–392. doi: 10.1162/jocn.1992.4.4.375, PMID: 23968130

[ref111] Paller K. A. Kutas M. McIsaac H. K. (1995). Monitoring conscious recollection via the electrical activity of the brain. Psychol. Sci. 6, 107–111. doi: 10.1111/j.1467-9280.1995.tb00315.x

[ref112] Patihis L. Otgaar H. Lynn S. J. Loftus E. F. McNally R. J. (2023). “The recovered memory debate: wins, losses, and creating future open-minded skeptics” in Toward a science of clinical psychology: A tribute to the life and works of Scott O. Lilienfeld. eds. Cobb C. L. Lynn S. J. O’Donohue W. (Berlin: Springer International Publishing), 377–394.

[ref113] Peláez Devesa M. Pérez Mata N. Diges Junco M. (2019). Influencia del conocimiento previo y la repetición de entrevistas: memoria y sugestión en una muestra de preescolares. Colombia Forense 6, 1–23. doi: 10.16925/2145-9649.2019.01.02

[ref114] Pérez-Mata N. Diges M. (2007). False recollections and the congruence of suggested information. Memory 15, 701–717. doi: 10.1080/09658210701647258, PMID: 17891682

[ref115] Pérez-Mata N. Diges M. Peláez M. (2022). Effects of divided attention and cued recall test on true and illusory memories in the DRM paradigm. Psicológica 43:e14569, 1–24. doi: 10.20350/digitalCSIC/14569

[ref116] Pérez-Mata M. N. Read J. D. Diges M. (2002). Effects of divided attention and word concreteness on correct recall and false memory reports. Memory 10, 161–177. doi: 10.1080/09658210143000308, PMID: 11958721

[ref117] Pergolizzi D. Chua E. F. (2015). Transcranial direct current stimulation (tDCS) of the parietal cortex leads to increased false recognition. Neuropsychologia 66, 88–98. doi: 10.1016/j.neuropsychologia.2014.11.012 25448859

[ref118] Peters M. J. van Oorsouw K. I. Jelicic M. Merckelbach H. (2013). Let's use those tests! Evaluations of crime-related amnesia claims. Memory 21, 599–607. doi: 10.1080/09658211.2013.771672 23425323

[ref119] Poldrack R. A. (2006). Can cognitive processes be inferred from neuroimaging data? Trends Cogn. Sci. 10, 59–63. doi: 10.1016/j.tics.2005.12.004 16406760

[ref120] Poldrack R. A. (2011). Inferring mental states from neuroimaging data: from reverse inference to large-scale decoding. Neuron 72, 692–697. doi: 10.1016/j.neuron.2011.11.001, PMID: 22153367 PMC3240863

[ref121] Polich J. (2007). Updating P300: an integrative theory of P3a and P3b. Clin. Neurophysiol. 118, 2128–2148. doi: 10.1016/j.clinph.2007.04.019, PMID: 17573239 PMC2715154

[ref122] Poole D. A. White L. T. (1991). Effects of question repetition on the eyewitness testimony of children and adults. Dev. Psychol. 27, 975–986. doi: 10.1037/0012-1649.27.6.975

[ref123] Porter S. Birt A. R. Yuille J. C. Lehman D. R. (2000). Negotiating false memories: interviewer and remember characteristics relate to memory distortion. Psychol. Sci. 11, 507–510. doi: 10.1111/1467-9280.00297, PMID: 11202498

[ref124] Principe G. F. Ceci S. J. (2002). "I saw it with my own ears": the effects of peer conversations on preschoolers' reports of nonexperienced events. J. Exp. Child Psychol. 83, 1–25. doi: 10.1016/S0022-0965(02)00120-0 12379416

[ref125] Principe G. F. Kanaya T. Ceci S. J. Singh M. (2006). Believing is seeing: how rumors can engender false memories in preschoolers. Psychol. Sci. 17, 243–248. doi: 10.1111/j.1467-9280.2006.01692.x 16507065

[ref126] Ranganath C. Paller K. A. (2000). Neural correlates of memory retrieval and evaluation. Cogn. Brain Res. 9, 209–222. doi: 10.1016/S0926-6410(99)00048-8 10729705

[ref127] Read J. D. (1996). From a passing thought to a false memory in 2 minutes: confusing real and illusory events. Psychon. Bull. Rev. 3, 105–111. doi: 10.3758/BF03210749, PMID: 24214811

[ref128] Reato D. Rahman A. Bikson M. Parra L. C. (2010). Low-intensity electrical stimulation affects network dynamics by modulating population rate and spike timing. J. Neurosci. 30, 15067–15079. doi: 10.1523/JNEUR-OSCI.2059-10.2010, PMID: 21068312 PMC3500391

[ref129] Roediger H. L. Balota D. A. Watson J. M. (2001). “Spreading activation and arousal of false memories” in The nature or remembering: Essays in honour of Robert G. Crowder. eds. Roediger H. L. Nairne J. S. Neath I. Surprenant A. M. (Washington, DC: American Psychological Association), 95–115.

[ref130] Roediger H. L. McDermott K. B. (1995). Creating false memories: remembering words not presented in lists. J. Exp. Psychol. Learn. Mem. Cogn. 21, 803–814. doi: 10.1037/0278-7393.21.4.803

[ref131] Rosenfeld J. P. Labkovsky E. (2010). New P300-based protocol to detect concealed information: resistance to mental countermeasures against only half the irrelevant stimuli and a possible ERP indicator of countermeasures. Psychophysiology 47, 1002–1010. doi: 10.1111/j.1469-8986.2010.01024.x, PMID: 20477980

[ref132] Rugg M. D. (1995). “ERP studies of memory” in Electrophysiology of mind: Event-related brain potentials and cognition. eds. Rugg M. D. Coles M. G. H. (Oxford: Oxford University Press), 133–170.

[ref133] Rugg M. D. Allan K. (2000). “Event-related potential studies of memory” in The oxford handbook of memory. eds. Tulving E. Craik F. I. M. (Oxford: Oxford University Press), 521–537.

[ref134] Rugg M. D. Cox C. J. C. Doyle M. C. Wells T. (1995). Event-related potentials and the recollection of low and high frequency words. Neuropsychologia 33, 471–484. doi: 10.1016/0028-3932(94)00132-9, PMID: 7617156

[ref135] Rugg M. D. Curran T. (2007). Event-related potentials and recognition memory. Trends Cogn. Sci. 11, 251–257. doi: 10.1016/j.tics.2007.04.004 17481940

[ref136] Rugg M. D. Walla P. Schloerscheidt A. M. Fletcher P. C. Frith C. D. Dolan R. J. (1998). Neural correlates of depth of processing effects on recollection: evidence from brain potentials and positron emission tomography. Exp. Brain Res. 123, 18–23. doi: 10.1007/s002210050540, PMID: 9835388

[ref137] Schacter D. L. (2022). The seven sins of memory: an update. Memory 30, 37–42. doi: 10.1080/09658211.2021.1873391, PMID: 33459149 PMC8285452

[ref138] Schacter D. L. Alpert N. M. Savage C. R. Rauch S. L. Albert M. S. (1996). Conscious recollection and the human hippocampal formation: evidence from positron emission tomography. Proc. Natl. Acad. Sci. 93, 321–325. doi: 10.1073/pnas.93.1.321, PMID: 8552630 PMC40230

[ref139] Schacter D. L. Buckner R. L. Koutstaal W. Dale A. M. Rosen B. R. (1997). Late onset of anterior prefrontal activity during true and false recognition: an event-related fMRI study. NeuroImage 6, 259–269. doi: 10.1006/nimg.1997.0305, PMID: 9417969

[ref140] Schacter D. L. Chamberlain J. Gaesser B. Gerlach K. D. (2012). “Neuroimaging of true, false, and imaginary memories” in Memory and law. eds. Nadel L. Sinnott-Armstrong W. P. (Oxford: Oxford University Press), 233–262.

[ref141] Schacter D. L. Slotnick S. D. (2004). The cognitive neuroscience of memory distortion. Neuron 44, 149–160. doi: 10.1016/j.neuron.2004.08.017 15450167

[ref142] Schooler J. W. Gerhard D. Loftus E. F. (1986). Qualities of the unreal. J. Exp. Psychol. Learn. Mem. Cogn. 12, 171–181. doi: 10.1037/0278-7393.12.2.171 2939174

[ref143] Schwarz N. Newman E. Leach W. (2016). Making the truth stick and the myths fade: lessons from cognitive psychology. Behav. Sci. Policy 2, 85–95. doi: 10.1353/bsp.2016.0009

[ref144] Scoboria A. Wade K. A. Lindsay D. S. Azad T. Strange D. Ost J.. (2017). A mega-analysis of memory reports from eight peer-reviewed false memory implantation studies. Memory 25, 146–163. doi: 10.1080/09658211.2016.1260747, PMID: 27892833

[ref145] Senkfor A. J. Van Petten C. (1998). Who said what? An event-related potential investigation of source and item memory. J. Exp. Psychol. Learn. Mem. Cogn. 24, 1005–1025. doi: 10.1037/0278-7393.24.4.1005, PMID: 9699305

[ref146] Sharman S. J. Scoboria A. (2009). Imagination equally influences false memories of high and low plausibility events. Appl. Cogn. Psychol. 23, 813–827. doi: 10.1002/acp.1515

[ref147] Sharman S. J. Scoboria A. (2011). Event plausibility and imagination inflation: a reply to Pezdek and Blandon-Gitlin. Appl. Cogn. Psychol. 25, 344–346. doi: 10.1002/acp.1705

[ref148] Smith R. E. (2003). The cost of remembering to remember in event-based prospective memory: investigating the capacity demands of delayed intention performance. J. Exp. Psychol. Learn. Mem. Cogn. 29, 347–361. doi: 10.1037/0278-7393.29.3.347, PMID: 12776746

[ref149] Stadler M. A. Roediger H. L. McDermott K. B. (1999). Norms for word lists that create false memories. Mem. Cogn. 27, 494–500. doi: 10.3758/BF03211543, PMID: 10355238

[ref150] Stark C. E. Okado Y. Loftus E. F. (2010). Imaging the reconstruction of true and false memories using sensory reactivation and the misinformation paradigms. Learn. Mem. 17, 485–488. doi: 10.1101/lm.1845710, PMID: 20861170

[ref151] Toglia M. P. Schmuller J. Surprenant B. G. Hooper K. C. DeMeo N. N. Wallace B. L. (2022). Novel approaches and cognitive neuroscience perspectives on false memory and deception. Front. Psychol. 13, 1–16. doi: 10.3389//fpsg.2022.721961 PMC8979290 35386904

[ref152] Tulving E. (1985). Memory and consciousness. Can. Psychol. 26, 1–12. doi: 10.1037/h0080017

[ref153] Tulving E. Osler S. (1968). Effectiveness of retrieval cues in memory for words. J. Exp. Psychol. 77, 593–601. doi: 10.1037/h0026069 5672271

[ref154] Tulving E. Thomson D. M. (1973). Encoding specificity and retrieval processes in episodic memory. Psychol. Rev. 80, 352–373. doi: 10.1037/h0020071

[ref155] Van de Ven V. Otgaar H. Howe M. L. (2017). A neurobiological account of false memories. in Finding the truth in the courtroom: Dealing with deception, lies, and memories. eds. Otgaar E. H. Howe M. L. (New York, Oxford Academic), 75–99.

[ref156] Volz K. Stark R. Vaitl D. Ambach W. (2019). Event-related potentials differ between true and false memories in the misinformation paradigm. Int. J. Psychophysiol. 135, 95–105. doi: 10.1016/j.ijpsycho.2018.12.002, PMID: 30527597

[ref157] Voss J. L. Paller K. A. (2009). Remembering and knowing: electrophysiological distinctions at encoding but not retrieval. NeuroImage 46, 280–289. doi: 10.1016/j.neuroimage.2009.01.048, PMID: 19457375

[ref158] Wagenaar W. A. Groeneweg J. (1990). The memory of concentration camp survivors. Appl. Cogn. Psychol. 4, 77–87. doi: 10.1002/acp.2350040202

[ref159] Wiese H. Daum I. (2006). Frontal positivity discriminates true from false recognition. Brain Res. 1075, 183–192. doi: 10.1016/j.brainres.2005.12.117, PMID: 16460706

[ref160] Wilding E. L. (2000). In what way does the parietal ERP old/new effect index recollection? Int. J. Psychophysiol. 35, 81–87. doi: 10.1016/S0167-8760(99)00095-1, PMID: 10683669

[ref161] Wilding E. L. Rugg M. D. (1996). An event-related potential study of recognition memory with and without retrieval of source. Brain 119, 889–905. doi: 10.1093/brain/119.3.889 8673500

[ref162] Wilding E. L. Rugg M. D. (1997a). Event-related potentials and the recognition memory exclusion task. Neuropsychologia 35, 119–128. doi: 10.1016/S0028-3932(96)00076-0 9025116

[ref163] Wilding E. L. Rugg M. D. (1997b). An event-related potential study of memory for words spoken aloud or heard. Neuropsychologia 35, 1185–1195. doi: 10.1016/S0028-3932(97)00048-1 9364489

[ref164] Wolk D. A. Sen N. M. Chong H. Riis J. L. McGinnis S. M. Holcomb P. J.. (2009). ERP correlates of item recognition memory: effects of age and performance. Brain Res. 1250, 218–231. doi: 10.1016/j.brainres.2008.11.014, PMID: 19046954 PMC2712353

[ref165] Wood J. M. Garven S. (2000). How sexual abuse interviews go astray: implications for prosecutors, police, and child protection services. Child Maltreat. 5, 109–118. doi: 10.1177/1077559500005002003, PMID: 11232084

[ref166] Ye Z. Zhu B. Zhuang L. Lu Z. Chen C. Xue G. (2016). Neural global pattern similarity underlies true and false memories. J. Neurosci. 36, 6792–6802. doi: 10.1523/JNEUROSCI.0425-16.2016, PMID: 27335409 PMC6601745

[ref167] Zhu B. Chen C. Shao X. Xue G. (2019). Multiple interactive memory representations underline the induction of false memory. Proc. Natl. Acad. Sci. USA 116, 3466–3475. doi: 10.1073/pnas.1817925116, PMID: 30765524 PMC6397536

